# Identifying disease-causing mutations with privacy protection

**DOI:** 10.1093/bioinformatics/btaa641

**Published:** 2020-07-19

**Authors:** Mete Akgün, Ali Burak Ünal, Bekir Ergüner, Nico Pfeifer, Oliver Kohlbacher

**Affiliations:** Translational Bioinformatics, University Hospital Tübingen, Tübingen 72026, Germany; Methods in Medical Informatics, Dept. of Computer Science, University of Tübingen, Tübingen 72026, Germany; Methods in Medical Informatics, Dept. of Computer Science, University of Tübingen, Tübingen 72026, Germany; CeMM Research Center for Molecular Medicine, Austrian Academy of Sciences, Vienna, Austria; Methods in Medical Informatics, Dept. of Computer Science, University of Tübingen, Tübingen 72026, Germany; Institute for Bioinformatics and Medical Informatics, University of Tübingen, Tübingen 72026, Germany; Statistical Learning in Computational Biology, Max Planck Institute for Informatics, Saarbrücken 66123, Germany; Translational Bioinformatics, University Hospital Tübingen, Tübingen 72026, Germany; Institute for Bioinformatics and Medical Informatics, University of Tübingen, Tübingen 72026, Germany; Applied Bioinformatics, Dept. of Computer Science, University of Tübingen, Tübingen 72026, Germany; Biomolecular Interactions, Max Planck Institute for Developmental Biology, Tübingen 72026, Germany

## Abstract

**Motivation:**

The use of genome data for diagnosis and treatment is becoming increasingly common. Researchers need access to as many genomes as possible to interpret the patient genome, to obtain some statistical patterns and to reveal disease–gene relationships. The sensitive information contained in the genome data and the high risk of re-identification increase the privacy and security concerns associated with sharing such data. In this article, we present an approach to identify disease-associated variants and genes while ensuring patient privacy. The proposed method uses secure multi-party computation to find disease-causing mutations under specific inheritance models without sacrificing the privacy of individuals. It discloses only variants or genes obtained as a result of the analysis. Thus, the vast majority of patient data can be kept private.

**Results:**

Our prototype implementation performs analyses on thousands of genomic data in milliseconds, and the runtime scales logarithmically with the number of patients. We present the first inheritance model (recessive, dominant and compound heterozygous) based privacy-preserving analyses of genomic data to find disease-causing mutations. Furthermore, we re-implement the privacy-preserving methods (MAX, SETDIFF and INTERSECTION) proposed in a previous study. Our MAX, SETDIFF and INTERSECTION implementations are 2.5, 1122 and 341 times faster than the corresponding operations of the state-of-the-art protocol, respectively.

**Availability and implementation:**

https://gitlab.com/DIFUTURE/privacy-preserving-genomic-diagnosis.

**Supplementary information:**

Supplementary data are available at *Bioinformatics* online.

## 1 Introduction

The advent of next-generation sequencing platforms has rapidly reduced the sequencing cost of individual genomes and made the genomic data an essential part of clinical research and diagnostics. It has been shown that monogenic rare disease cases can greatly benefit from whole genome or whole exome sequencing in both the research and the clinical settings. Although there are a vast number of rare disease patients, reaching up to 10% of the population in some regions, it is challenging to find patients with the same phenotype due to the dispersion of cases to more than 7000 diseases (https://www.omim.org/statistics/entry, https://www.who.int/genomics/public/geneticdiseases). This is important in the research setting where the causative gene is not known and more cases with the same phenotype are needed for further analysis. In the clinical setting, it has been shown that having genomic data of multiple individuals from the same family, such as trio sequencing, greatly improves the diagnostic yield (Need *et al.*, 2012; Retterer *et al.*, 2016; Sanders *et al.*, 2012; Wang *et al.*, 2019). Evidently, sharing and comparison of many individuals’ genomic data are crucial for rare disease cases; however, this might also bring a significant compromise in genomic privacy since rare disease studies require access to the rare and personal variants in the genome. Therefore, it is crucial to a have platform that provides functions, enabling rare disease studies with a high level of privacy protection.

Sharing of genome data maximizes the benefit from existing datasets and aims to increase research efficiency. Sharing all sequenced genomes will increase the success of disease–gene association studies. Several global sharing platforms have been developed for cancer and rare disease researches by The International Cancer Genome Consortium (ICGC) (2010), the Cancer Genome Atlas (TCGA) (https://www.cancer.gov/tcga), the International Rare Diseases Research Consortium (IRDiRC) (Cutillo *et al.*, 2017) and, the Global Alliance for Genomics and Health (GA4GH) (2016). However, large-scale data-sharing approaches have achieved limited success due to the risk of re-identifying participants (Clayton, 2010; Homer *et al.*, 2008; Jacobs *et al.*, 2009; Sankararaman *et al.*, 2009; Visscher and Hill, 2009) so individuals having genetic disease phenotypes will be particularly resistant to sharing of such information because of fear of discrimination and prejudices. For example, Shringarpure and Bustamante (Shringarpure and Bustamante, 2015) showed that the beacons in the Beacon Network (Cutillo *et al.*, 2017) are vulnerable to an attack in which an adversary having only a small portion of a patient’s genome can re-identify the anonymous patient whose genome data are shared in the Beacon Network. Re-identification can be done using the background information that comes with public DNA sequences (Gymrek *et al.*, 2013). Another example is the identification of the Personal Genome Project participants using general demographic data (Sweeney *et al.*, 2013). Furthermore, it is possible to identify individuals that did not undergo genetic testing before with the genome-wide genotyping profile obtained from their genetic relatives’ DNAs (Ellenbogen and Narayanan, 2019; Erlich *et al.*, 2018).

### 1.1 Related work

A hardware-based solution for privacy-protected rare disease analysis has been proposed in the study by Chen *et al.* (2017). In this work, Intel SGX is used to perform reliable calculations on genomic data. Transmission disequilibrium test (TDT), which is a family-based relationship test for the presence of a genetic link between the genetic marker and the trait, is needed for rare disease analysis. Since SGX is a limited device in terms of memory, it is not possible to process the whole data at the same time. For this reason, genomic data are sent to the SGX after fragmentation, and the top *N* single-nucleotide polymorphism (SNP) values are retained in a global queue. The data coming to the SGX is decrypted, decompressed and then used for TDT analysis.

Wang *et al.* (2017) proposed a solution based on differential privacy for TDT based on test statistics, *P*-values and the shortest Hamming distance scores. The privacy of an entire family participating in the study is considered as the target of the attacker. The proposed solution only works for families having a single child, which is a major drawback. Furthermore, it does not perform privacy-preserving TDT on correlated SNPs.

Jagadeesh *et al.* (2017) proposed a solution based on secure multi-party computation (MPC) for protecting participants’ privacy in genomic diagnosis. They applied their solution to three real-life scenarios which are small patient cohorts (MAX), trio analysis (SETDDIFF) and two-hospital collaboration (INTERSECTION). The proposed solution performs analyses while keeping all variants and genes involved in the computation private. In the end, only candidate genes and variants are disclosed as output. In their study, Jagadeesh *et al.* did not use pedigree and genotype information, which allow the filtering of variants that fit inheritance patterns.

### 1.2 Our contributions

In this study, we propose a solution for privacy-preserving rare disease analysis via secure MPC. We show how to protect individuals’ privacy while analysing their genomic data in the presence of different inheritance models (recessive, dominant and compound heterozygous) to find possible disease-causing variants. Our solution also enables privacy-preserving cross-institutional collaborations for rare disease analysis. To this end, we propose secure protocols based on the combination of arithmetic and Boolean sharing in the same computation and try to evaluate the operations that have an efficient representation as an arithmetic circuit or Boolean circuit. As stated in the study by Demmler *et al.* (2015), conversion from arithmetic shares to Boolean shares is a non-linear operation and costly in terms of communication and computation. Our solution does not need to use the non-linear conversion from arithmetic shares to Boolean shares. Thus, we perform a mixed MPC protocol execution with low communication cost and computation cost, and increase the performance of the overall process noticeably. Furthermore, we significantly improve the promising methods proposed by Jagadeesh *et al.* (2017). Our implementation provides much better runtimes than Jagadeesh *et al.*’s (2017) solution and makes it possible to work on thousands of patient data and millions of variants. We also show that our methods provide more accurate results than the methods in the study by Jagadeesh *et al.* (2017). To the best of our knowledge, our methods are the first providing privacy-preserving analysis of large-scale genomic data under genetic inheritance models to find disease-causing mutations.

## 2 Materials and methods

In this section, we introduce preliminaries, the proposed privacy-preserving methods and security considerations.

### 2.1 Frequency-based filters for diagnosing monogenic diseases

Personalized genomics is used to diagnose many monogenic diseases (Wenger *et al.*, 2017). Frequency-based filters in which patient genomes are compared with as many genomes as possible are extremely successful at the diagnosis of monogenic diseases (Rehm *et al.*, 2013). Jagadeesh *et al.* (2017) defined three frequency-based filters (MAX, INTERSECTION and SETDIFF). MAX is used to find a gene with rare functional mutations in a large number of affected individuals. INTERSECTION finds rare functional mutations shared by all individuals in a cohort. SETDIFF finds rare functional mutations shared by all affected individuals but not seen in any unaffected individual.

In this study, we use family information to find the variants fitting three autosomal Mendelian inheritance models, namely recessive, dominant and compound heterozygous. In case of recessive inheritance, both parents of the affected patient should be heterozygous while the patient is homozygous for the causative variant. If the disorder is autosomal dominant, the affected individuals from the same family should all be heterozygous. In compound heterozygosity, the affected person should have at least two distinct heterozygous variants on the causative gene which are inherited from the different parents.

### 2.2 Secure MPC

Secure MPC was proposed in the early 1980s (Goldreich *et al.*, 1987; Yao, 1986). These studies show that multiple parties can compute any function on inputs without learning anything about the inputs of the other parties. Let us assume that there are *n* parties $I_{1},\ldots ,I_{n}$. The *I_i_* has a private input *x_i_*. All parties want to compute the arbitrary function $\left(y_{1},\ldots ,y_{n})=f(x_{1},..,x_{n}\right)$ and get the result *y_i_*. MPC allows the parties to compute the function through an interactive protocol and allows *I_i_* to learn only *y_i_*.

The security of an MPC protocol is preserved even in the presence of some adversaries that corrupt some of the participating parties, modify transcripts and mimic their behaviour. There are two types of adversaries. Semi-honest adversaries follow the protocol definition but try to learn the secret information from the messages that they obtained during the protocol execution. Malicious adversaries, on the other hand, may deviate from the protocol definition to learn secret information.

MPC calculations may require many interaction rounds and large data conversions between parties. For these reasons, it may be very difficult to implement MPC in practice. Some successful works have been done to reduce the complexity of MPC and to implement it in practice for some problems (Bogdanov *et al.*, 2008; Demmler *et al.*, 2015; Huang *et al.*, 2011; Liu *et al.*, 2015; Malkhi *et al.*, 2004). The schemes proposed in these studies allow us to utilize the benefits of MPC for some real-life applications.

An important building block of MPC is oblivious transfer (OT) (Rabin, 1981). OT is a protocol where a sender transmits one of the many pieces of information to a receiver but remains unaware of which piece is transmitted. In 1-out-of-2 OT, the sender inputs two *l*-bit strings (*s*_0_, *s*_1_) and the receiver inputs a bit c∈{0,1}. At the end, the receiver receives *s_c_* and learns no information about $s_{1-c}$, and the sender learns no information about *c*.

In the rest of the article, we denote a shared value *x* as ${\langle x\rangle }^{t}$ where t∈{A=Arithmetic,B=Boolean} indicates the sharing type.

#### 2.2.1 Arithmetic sharing

In arithmetic sharing, an *l*-bit value *x* is shared additively in the ring ${\mathbb{Z}}_{2^{l}}$ as the sum of two values. For example, ${\mathbb{Z}}_{4}=\{\bar{0},\bar{1},\bar{2},\bar{3}\}$ and the sum $\bar{x}+\bar{y}$ in ${\mathbb{Z}}_{4}$ are the remainder when the integer *x *+* y* is divided by 4. For *l*-bit secret sharing of *x*, we have ${\langle x\rangle }_{0}^{A}+{\langle x\rangle }_{1}^{A}\equiv x(\;\mathrm{mod}\;2^{l})$ where *I_i_* knows only ${\langle x\rangle }_{i}^{A}$ and i∈{0,1}. All arithmetic operations are performed in the ring ${\mathbb{Z}}_{2^{l}}$. For arithmetic sharing, we use protocols based on Beaver’s multiplication triples (Beaver, 1991).

**Addition.** ${\langle z\rangle }^{A}={\langle x\rangle }^{A}+{\langle y\rangle }^{A}$. *I_i_* locally computes ${\langle x\rangle }_{i}^{A}+{\langle y\rangle }_{i}^{A}$. To compute the addition of a shared value ${\langle x\rangle }^{A}$ and a constant *c*, *I_i_* locally computes ${\langle z\rangle }_{i}^{A}={\langle x\rangle }_{i}^{A}+c$ and $I_{1-i}$ locally computes ${\langle z\rangle }_{1-i}^{A}={\langle x\rangle }_{1-i}^{A}$

**Multiplication.** ${\langle z\rangle }^{A}={\langle x\rangle }^{A}\cdot {\langle y\rangle }^{A}$. Multiplication is performed using a pre-computed multiplication triple ${\langle c\rangle }_{i}^{A}={\langle a\rangle }_{i}^{A}\cdot {\langle b\rangle }_{i}^{A}$ (Beaver, 1991). The computation of the multiplication triple is performed via homomorphic encryption or OT. *I_i_* cannot perform multiplication locally. More details can be found in the study Demmler *et al.* (2015).

#### 2.2.2 Boolean sharing

In Boolean sharing, an *l*-bit value *x* is shared using an XOR-based sharing scheme as the XOR of two values. For *l*-bit secret sharing of *x*, we have ${\langle x\rangle }_{0}^{B}⊕{\langle x\rangle }_{1}^{B}=x$ where ${\langle x\rangle }_{0}^{B},{\langle x\rangle }_{1}^{B}\in {\mathbb{Z}}_{2}$ and *I_i_* knows only ${\langle x\rangle }_{i}^{B}$ where i∈{0,1}. For Boolean sharing, we use the protocol of Goldreich–Micali–Wigderson (GMW) (Goldreich *et al.*, 1987).

**XOR.** ${\langle z\rangle }^{B}={\langle x\rangle }^{B}⊕{\langle y\rangle }^{B}$. *I_i_* locally computes ${\langle x\rangle }_{i}^{B}⊕{\langle y\rangle }_{i}^{B}$.

**AND.** ${\langle z\rangle }^{B}={\langle x\rangle }^{B}\wedge {\langle y\rangle }^{B}$. AND is performed using a pre-computed Boolean multiplication triple ${\langle c\rangle }_{i}^{B}={\langle a\rangle }_{i}^{B}\wedge {\langle b\rangle }_{i}^{B}$. *I_i_* cannot perform AND locally. A Boolean multiplication triple is pre-computed efficiently using random OT (R-OT) in the offline phase. More details can be found in the studies by Asharov *et al.* (2013) and Demmler *et al.* (2015).

### 2.3 Mixed protocol execution

The main purpose of the execution of the mixed secure protocol is to combine operations that are carried out efficiently in different protocols in a single secure application [e.g. the usage of the efficient operations of arithmetic sharing (additions and multiplications) and the efficient operations of Boolean sharing (comparisons and multiplexers) in a single secure application]. In this study, we try to reduce the communication load and the execution time of secure computation as much as possible. In this context, we want to combine efficient operations of arithmetic and Boolean sharing in our methods.

In our study, all secret information is shared using arithmetic sharing. Since addition is carried out locally in arithmetic sharing, we use it to find the locations of variants that are present in either all or none of a group of individuals. These locations are marked with zero by subtraction in arithmetic sharing. We use the logical OR and comparison operations in Boolean sharing to mark the found locations with one and mark all other locations with zero. We need to convert the arithmetic shares to Boolean shares. However, converting arithmetic shares to Boolean shares cannot be done locally and requires high communication and computation cost (Demmler *et al.*, 2015). In our methods, the locations of variants that the researcher wants to find are marked with zero, and we can convert arithmetic-shared zero values to Boolean-shared zero values locally without any communication between two parties (see Section 2.4).

### 2.4 Equality test for arithmetic-shared zero values without bit decomposition in two-party computation

Conversion of arithmetic shares to Boolean shares is an expensive operation in secure MPC. Share conversion is a non-linear process and requires a lot of communication and evaluation of cryptographic operations. As the size of the processed data increases, computation time and communication cost of conversion also increase. On the other hand, it is possible to convert the arithmetic-shared zero values to the Boolean-shared zero values without bit decomposition in the two-party setting. This conversion is done by taking the arithmetic inverse of shares in one of two parties. As illustrated inFigure 1 for *l *=* *4, it works correctly only for zero values which is only what we are interested in and may produce different results for non-zero values. Thus, we can compare arithmetic-shared zero values with constant zero values known by both parties without converting them to Boolean shares using bit decomposition. The detailed description of our linear conversion method is given in Supplementary Algorithm S1.

**Fig. 1.**
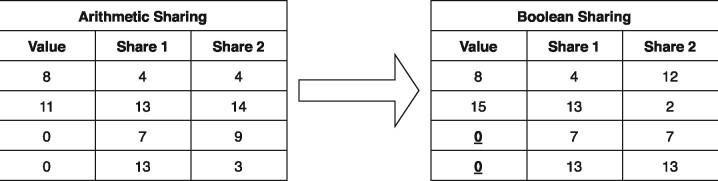
A method for converting arithmetic-shared zero values to Boolean-shared zero values without bit decomposition. The conversion is performed by inverting Share two values. This local conversion only works for zero values. The bit-length *l* is 4

### 2.5 Representation of genomic data

**Fig. 2.**
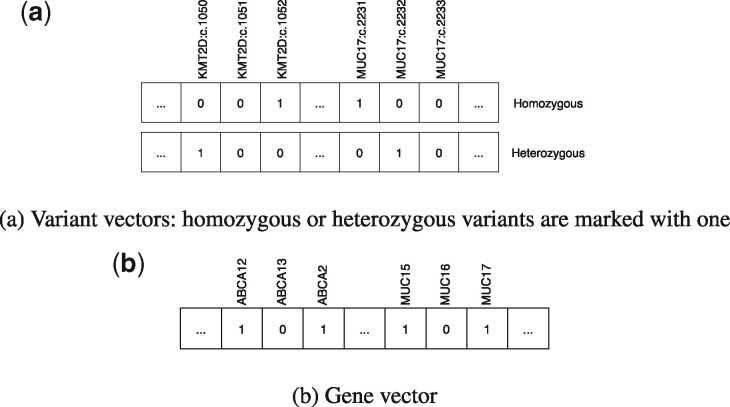
Individuals create a gene vector and two variant vectors

To be amenable to MPC as described above, each individual’s genotype has to be encoded in a bit vector representation. Obviously, there are many different possibilities for encoding this information. In line with previous efforts (Jagadeesh *et al.*, 2017), we chose to represent missense and non-sense variants only. More complex encodings are, however, possible in principle and the method is fundamentally not limited to this representation. To be able to distinguish homozygous and heterozygous variants, we extended the model by Jagadeesh *et al.* In this work, we represent around 28 million variants of the human genome by two bit vectors (Fig. 2), representing homozygous and heterozygous variants, respectively. We also represent all genes of the human genome by a bit vector (Fig. 2). Genes carrying one or more rare functional variants are marked with 1 in the bit vector.

### 2.6 System overview

**Fig. 3.**
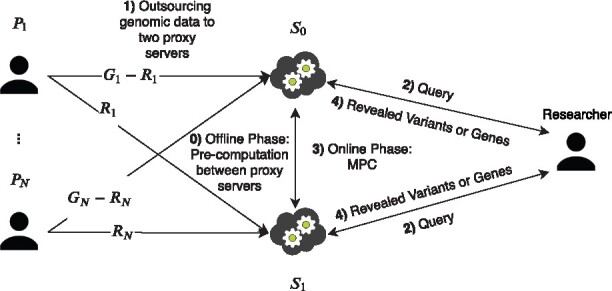
General system architecture of our solution. Patients $P_{1}\ldots P_{N}$ communicate with two non-colluding proxy servers *S*_0_ and *S*_1_. Researchers can perform RECESSIVE, DOMINANT, COMPHET, INTERSECTION, SETDIFF and MAX operations on all data through these proxy servers in a secure manner

In the proposed system, there are three types of participants: patients $P_{1},P_{2},\ldots ,P_{N}$ having vectors of genes and variants $G_{1},G_{2},\ldots ,G_{N}$, respectively, two non-colluding proxy servers *S*_0_ and *S*_1_, and researchers. Patients split their variant vectors and gene vectors into two arithmetic shares and send them to the two proxy servers. All genome data are hidden from the two proxy servers. This closely matches the cloud computing paradigm where computing and data storage are outsourced to powerful machines provided by an external party. Our system is based on the outsourcing protocol of Kamara and Raykova (2011). They give a general architecture and security proof to convert the *N*-party MPC protocol into a secure protocol where the data are outsourced to *M* non-colluding servers. A similar architecture was used in several studies (Asharov *et al.*, 2017; Demmler *et al.*, 2017; Jagadeesh *et al.*, 2017; Schneider and Tkachenko, 2018; Tkachenko *et al.*, 2018). After the two proxy servers receive a shared gene vector and two variant vectors from each participating patient, they use a secure two-party computation protocol to compute a function of interest. The architecture of the proposed system is described in Figure 3. In a real-world setting, these two servers can be managed by different government organizations. We assume that individuals have private access to their genomes. Furthermore, hospitals or biobanks collecting individuals’ genome can also outsource their storage and computational load to two non-colluding servers.

### 2.7 Protocol description

In this section, we describe the phases of our protocol and depict it in Figure 3.

**Phase 0—Offline Phase:** The offline phase which is independent of private inputs can be computed at any point before MPC computing (Phase 3) takes place. As explained in Sections 2.2.1 and 2.2.2, multiplication triples are required in arithmetic and Boolean sharing. In the offline phase, the proxy servers calculate multiplication triples independently of private inputs. The expensive parts of MPC are shifted into the offline phase which calculates multiplication triples based on OT. The proxy servers just need to know the size of the actual private inputs to calculate multiplication triples in the offline phase. In our solution, the length of gene and variant vectors are known by the proxy servers.

**Phase 1—Outsourcing:** As described in Section 2.5, a patient *P_i_* generates a vector of homozygous variants $V_{i}^{H}$, a vector of heterozygous variants $V_{i}^{E}$ and a gene vector $V_{i}^{G}$ from his or her genome. Due to privacy concerns, *P_i_* generates a random mask $R_{i}^{H}$ of the size of $V_{i}^{H}$, a random mask $R_{i}^{E}$ of the size of $V_{i}^{E}$ and a random mask $R_{i}^{G}$ of the size of $V_{i}^{G}$. *P_i_* sends $R_{i}^{T}$ to *S*_1_ and $\left(V_{i}^{T}-R_{i}^{T}\right)$ to *S*_0_ where T∈{H,E,G}. This ensures that the genome data are protected against the two non-colluding servers. This phase is carried out only once. Analyses can be performed on secret shared genomic data multiple times.

**Phase 2—Researcher Query:** A researcher sends the list of identifiers of individuals participating in the analyses to each proxy server. He also sends the type of analysis, and pedigree information if needed.

**Phase 3—MPC Online Phase:** The proxy servers *S*_0_ and *S*_1_ run the private DOMINANT, RECESSIVE, COMPHET, MAX, SETDIFF, or INTERSECTION protocol using MPC on the genomic data of individuals whose identifiers are given in the researcher’s query. After the MPC protocol execution, *S*_0_ and *S*_1_ hold a secret share of the output vector.

**Phase 4—Output Reconstruction:** The proxy servers *S*_0_ and *S*_1_ hold one share of the output vector. *S*_0_ and *S*_1_ send their shares to the researcher. The researcher computes the XOR of the shares and extracts the plaintext vector representing the candidate variants or genes.

### 2.8 Privacy-preserving RECESSIVE operation

In the RECESSIVE operation, we find the locations in which all affected siblings have homozygous rare variants, and the parents have heterozygous rare variants by getting the summation of the homozygous variant vectors of all affected siblings and heterozygous variant vectors of the parents. Assume that the number of affected siblings is *n*, the locations found are marked with *n *+* *2. To mark the locations found with zero in a vector ${\langle a\rangle }_{t}^{A}$, a vector filled with *n *+* *2 is subtracted from ${\langle a\rangle }_{t}^{A}$. The locations in which all unaffected siblings have non-homozygous variants and all non-family individuals do not have variants are found by getting the summation of heterozygous and homozygous variant vectors of all non-family individuals and homozygous variant vectors of all un-affected siblings. The locations found are marked with zero in a vector ${\langle o\rangle }_{t}^{A}$. Our local conversion method is applied to ${\langle a\rangle }_{t}^{A}$ and ${\langle o\rangle }_{t}^{A}$; and, ${\langle a\rangle }_{t}^{B}$ and ${\langle o\rangle }_{t}^{B}$ are obtained, respectively. All transactions performed so far are carried out locally and without any communication between the two proxy servers. The locations we want to find in ${\langle a\rangle }_{t}^{B}$ and ${\langle o\rangle }_{t}^{B}$ are marked with zero. To mark zero-marked locations with one and all other locations with zero, ${\langle a\rangle }_{t}^{B}$ and ${\langle o\rangle }_{t}^{B}$ are compared to a vector filled with zeros using a Boolean equality gate which is evaluated by the GMW protocol. The common locations marked with one in ${\langle a\rangle }_{t}^{B}$ and ${\langle o\rangle }_{t}^{B}$ are found by taking logical AND of them. The pseudo-code of the RECESSIVE operation is given in Algorithm 1.

**Algorithm 1: Secure Recessive Operation**

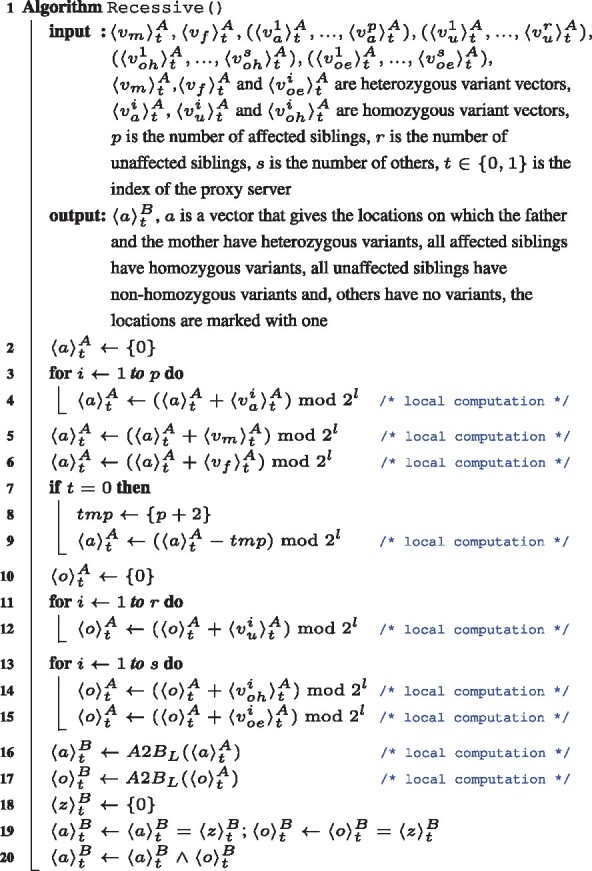

### 2.9 Privacy-preserving DOMINANT operation

In the DOMINANT operation, we find the locations in which all affected siblings have heterozygous variants. Assume that the number of affected siblings is *n*, the locations are marked with *n* in a vector ${\langle a\rangle }_{t}^{A}$. We mark these locations with zero by subtracting a vector filled with *n* from ${\langle a\rangle }_{t}^{A}$. The locations in which all unaffected siblings and all non-family individuals have homozygous reference variants are found by getting the summation of heterozygous and homozygous variant vectors of all unaffected siblings and non-family individuals. The locations found are marked with zero in a vector ${\langle o\rangle }_{t}^{A}$. We obtain two vectors ${\langle a\rangle }_{t}^{A}$ and ${\langle o\rangle }_{t}^{A}$ as we obtain in the RECESSIVE operation. The next steps are exactly the same as the steps in the RECESSIVE operation. The pseudo-code of the DOMINANT operation is given in the Supplementary Material.

### 2.10 Privacy-preserving COMPHET operation

In the COMPHET operation, we find the locations in which all affected siblings and the mother have non-heterozygous variants, the locations in which all affected siblings and the father have non-heterozygous variants, and the locations in which all unaffected siblings and non-family individuals have non-heterozygous variants are found by getting the summation of variant vectors. These locations are marked with zero in vectors ${\langle m\rangle }_{t}^{A},\;{\langle f\rangle }_{t}^{A}$ and ${\langle o\rangle }_{t}^{A}$, respectively. These vectors are computed with local operations that do not require communication between the two proxy servers. Our local conversion method is applied to ${\langle m\rangle }_{t}^{A},\;{\langle f\rangle }_{t}^{A}$ and ${\langle o\rangle }_{t}^{A}$; and, ${\langle m\rangle }_{t}^{B},\;{\langle f\rangle }_{t}^{B}$ and ${\langle o\rangle }_{t}^{B}$ are obtained, respectively. We mark the locations of zeros in ${\langle m\rangle }_{t}^{B},\;{\langle f\rangle }_{t}^{B}$ and ${\langle o\rangle }_{t}^{B}$ with one using a Boolean equality gate which is evaluated by the GMW protocol. By evaluating the Boolean AND gate a few times, the operation outputs the vectors ${\langle m\rangle }_{t}^{B}$ and ${\langle f\rangle }_{t}^{B}$. ${\langle m\rangle }_{t}^{B}$ is the set of heterozygous variants that appear exclusively on the mother and transmitted to all affected siblings and ${\langle f\rangle }_{t}^{B}$ is the set of heterozygous variants that appear on only the father and transmitted to all affected siblings. For every gene, we find the Cartesian product of two sets of heterozygous variants, more specifically the sets of indices of marks in vectors ${\langle m\rangle }_{t}^{B}$ and ${\langle f\rangle }_{t}^{B}$, respectively.

In other privacy-preserving operations, we find the locations of variants that are present either in all or none of a group of individuals using methods similar to the method we describe above. After these locations are found, arithmetic-shared zero values are converted to Boolean-shared zero values with our conversion method. The result vector is then generated using effective operations in Boolean sharing. The commented pseudo-codes of all secure operations are given in the Supplementary Material.

### 2.11 Security considerations

We discuss the security of our scheme in this section. The main purpose of our protocol is to provide privacy for patients whose data are outsourced to our service. The privacy of our scheme is based on the proven security of the GMW protocol (Goldreich *et al.*, 1987) and the protocol based on Beaver’s multiplication triples (Beaver, 1991). We assume that the two proxy servers used for secure computation do not collude. Genome data are shared between the two servers using arithmetic sharing and converted to Boolean sharing during the protocol execution. A semi-honest adversary corrupting at most one of the two proxy servers can observe a share of patients’ data. As the data are shared with arithmetic sharing or Boolean sharing, it looks like uniformly distributed random data and this prevents the leakage of the patients’ data.

The researcher specifies a list of patients whose data will be used in the analysis. If the researcher has permission for the relevant patients’ data, they are included in the analysis. The variants or genes obtained at the end of the analysis should be disclosed to the researcher. Unrelated individuals in a cohort cannot learn anything about each other more than their shared causal gene (MAX operation). In trio analysis, the researcher only learns causal variants of the affected individual and cannot learn any definite information about the parents or the control individuals (RECESSIVE, DOMINANT, COMPHET and SETDIFF operations). In the INTERSECTION operation, the researcher only learns the common variants of participating individuals. For this reason, Jagadeesh *et al.* (2017) have defined a protection quotient which is the fraction of the number of the variants or genes that are not exposed to the researcher to the total number of the variants or genes.

Confidentiality, integrity and, authentication between all parties are provided using state-of-the-art technologies such as TLS (Dierks and Rescorla, 2008).

## 3 Implementation

We have implemented the prototype of the proposed solution using the C++ programming language and the ABY framework (Demmler *et al.*, 2015), which provides an efficient implementation of secure two-party computation protocols. This framework works like a virtual machine that abstracts secure computational protocols. We use the implementation of the GMW protocol in the ABY framework to perform the non-linear parts of the operations in our protocol. A function is represented as an arithmetic or Boolean circuit. There are two primitive gates operations: linear gates (addition in arithmetic circuits, XOR in Boolean circuits) and non-linear gates. The execution of linear gates does not require communication and the evaluation of cryptographic operations. Hence, the performance of securely evaluating a function can be improved by reducing the number of non-linear gates. In the ABY framework, the total execution is divided into two phases: offline and online. In the offline phase, multiplication triples are pre-computed. The online phase that uses pre-computed multiplication triples to compute the function in the parties’ private inputs is very effective.

We implemented our methods using mixed protocol execution. We use both arithmetic and Boolean circuits in our implementation. We realized our novel linear method of converting arithmetic-shared zero values to Boolean-shared zero values (see Section 2.4). In our implementation, we benefited from the execution speed of this method. In Table 1, we compare our methods implemented with both arithmetic and Boolean gates with the methods implemented using only Boolean gates in terms of the number of linear and non-linear gates. It shows that our methods perform much better by decreasing the number of non-linear gates. To improve circuit evaluation time, we use SIMD (single instruction on multiple data) gates. The inputs to secure computation are vectorized. Each operation in the secure computation processes the vectorized input of the same length. For this reason, regardless of the input length, we give the number of operations in Table 1.

**Table 1.** Comparison of our privacy-preserving methods with the privacy-preserving methods implemented using Boolean circuits

Operation	Boolean AND	Boolean OR	Boolean INV	Boolean ADD	Boolean EQ	Boolean MUX	Boolean GT	Arithmetic ADD	Arithmetic to Boolean conversion
Boolean RECESSIVE	*p* + 2	2s+r−1	1	0	0	0	0	0	0
Boolean DOMINANT	*p*	r+s−1	1	0	0	0	0	0	0
Boolean COMPHET	*p* + 5	r+s−1	3	0	3	0	0	0	0
Boolean MAX	0	0	0	*n* – 1	0	*g* – 1	*g* – 1	0	0
Boolean INTERSECTION	*n* – 1	*n*	0	0	0	0	0	0	0
Boolean SETDIFF	1	2p+2r+2	1	0	0	0	0	0	0
Mixed RECESSIVE	1	0	0	0	2	0	0	p+r+2s+1	2
Mixed DOMINANT	1	0	0	0	2	0	0	p+2r+2s−1	2
Mixed COMPHET	6	2	2	0	3	0	0	p+r+s+2	3
Mixed MAX	1	0	0	0	0	*g* – 1	*g* – 1	*n* – 1	1
Mixed INTERSECTION	0	0	0	0	1	0	0	2*n*	1
Mixed SETDIFF	1	0	0	0	2	0	0	2p+2r+3	2

*Note:* The number of non-linear Boolean AND and OR operations in all methods implemented using Boolean circuits increase linearly with the number of participants. In our methods implemented using both arithmetic and Boolean circuits, the number of linear arithmetic ADD operation increases linearly with the number of participants and the numbers of all non-linear Boolean operations are fixed and independent from the number of participants. In addition, we use our linear method for converting arithmetic shared zero values to Boolean-shared zero values. Thus our methods work faster.

*p*, number of affected siblings; *r*, number of unaffected siblings; *s*, number of others; *n*, number of participants; *g*, number of genes.

We perform all operations on *l*-bit values where *l *=* *32. This means the ring of arithmetic sharing is ${\mathbb{Z}}_{2^{32}}$. In arithmetic sharing, we use only the addition operation that is run by two servers locally. This implies that our implementation does not require an offline phase, in which multiplication triples are generated, for operations in arithmetic sharing. Our solution requires Boolean multiplication triples, which are computed in the offline phase using OT, to evaluate Boolean ANDs. The cost of the offline phase scales linearly with the bit-length *l* as shown in Supplementary Tables S1–S3, S5, S7, S8 and S11.

## 4 Results

We evaluated the performance of the proposed solution both on real and synthetic data. We reported the performance of the online and offline phases of our protocol separately. We conducted our experiments in two Amazon EC2 instances (M4.2xlarge). Each instance ran an 8-core 2.4 GHz Intel Xeon E5-2676 v3 (Haswell) processor and had 32 GB of memory. We used a wide-area network (WAN) setting where the two servers are far apart. We placed one of the servers in Frankfurt and the other in London. We ran experiments on Ubuntu 16.04 with 4.13.0-36-generic kernel, compiled our implementation with gcc v5.4.0 and used a symmetric security level of 128 bits. The bandwidth was limited to 10Mbps and the average round trip time is 13.64 ms. For all timing results, each experiment has been repeated 20 times, and the average execution times have been reported. We use an additional communication setting to evaluate the impact, which is a local-area network (LAN) setting where two identical computers that are connected via gigabit ethernet are used. Each computer has a 3.20 GHz Intel i5-6500 CPU and 32 GB RAM. We give the detailed results of the experiments in the Supplementary Material.

### 4.1 Experiments on real data

We tested our algorithm on whole exome data of 6 patients with cerebrofaciothoracic dysplasia (Alanay *et al.*, 2014). We used VEP (McLaren *et al.*, 2016) for annotating the raw variant data which were stored in VCF (v4.1) format. VEP was configured to output every annotation available in the Esembl (release 91) for the hg19 human assembly. The most deleterious outcome for each variant was selected by enabling the VEP’s pick option. To identify the variants which will be used to calculate the MAX value, we developed an in-house script to select the variants which have less than 1% population allele frequency in any of the sub-population defined in the gnomAD (Exome Aggregation Consortium, 2016) and have HIGH or MODERATE impact on the protein coding genes. We also used an in-house exome cohort (*n* = 182) to remove any population specific polymorphisms and false-positive variant calls. We only considered the variants which were homozygous in the patients for operations because their families were consanguineous and the disorder was reported to be autosomal recessive. To identify the variants which will be used to calculate the RECESSIVE, SETDIFF and INTERSECTION operations, we selected the variants which have less than 5% population allele frequency and have HIGH or MODERATE impact on the protein coding genes. We used filter_vep (McLaren *et al.*, 2016) and VCF Explorer (Akgün and Demirci, 2017) for filtering the variants. Alanay *et al.* (2014) reported TMCO1 mutation as the cause of cerebrofaciothoracic dysplasia. In this study, disease-causing variants were reported recessive so we executed only our secure RECESSIVE operation on the data of this study. We made computations on variant vectors with a length of 28 000 000 and gene vectors with a length of 20 633. The details of the experiment results are given in Tables 2–5.

**Table 2.** Results of secure RECESSIVE operations on real patient data

T	Family member	#Rare variants	#Revealed variants	Gene name^a^	Protection quotient	Offline phase		Online phase	
						Comm. (MiB)	Time (ms)	Comm. (MiB)	Time (ms)
1	M	860	12	N/R	1-36/2880=98.7	4480	58 347	74	1135
	F	967	12	N/R					
	P	1053	12	**TMCO1**					
				MUC12					
				UBXN11					
2	M	1062	7	N/R	1-21/3003=99.3	4480	57 792	74	1088
	F	906	7	N/R					
	P	1035	7	**TMCO1**					
				MUC12					

*Note*: Our analysis reveals recessive mutations on TMCO1 gene which are causative mutations of cerebrofaciothoracic dysplasia.

a Proven causal gene name is highlighted.

M, Mother; F, Father; P, Proband; T, Trio.

**Table 3.** Results of secure MAX operation on real patient data

#Patient	#Rare variants (genes) per patient (median)	#Patient with rare variant in top three gene	Gene name^a^	Protection quotient	Offline phase		Online phase	
					Comm.(MiB)	Time (ms)	Comm. (MiB)	Time (ms)
4	228 (212)	4	MUC12	1-4/857=99.5	10	267	12	129
		3	ATXN3					
		2	**TMCO1**					

Note: We analysed a small cohort of four unrelated individual. These individuals are two of affected children suffering from cerebrofaciothoracic dysplasia (Alanay *et al.*, 2014). Our secure MAX operation reveals TMCO1 as the third most mutated gene among patients in a cohort. TMCO1 is the proven causal gene of cerebrofaciothoracic dysplasia.

a Proven causal gene name is highlighted.

**Table 4.** Results of secure SETDIFF operations on real patient data

T	Family member	#Rare variants	#Revealed variants	Protection quotient	Offline phase		Online phase	
					Comm. (MiB)	Time (ms)	Comm. (MiB)	Time (ms)
1	M	860	N/R	1-385/2880=86.6	896	11 282	18	317
	F	967	N/R					
	P	1053	385					
2	M	1062	N/R	1-336/3003=88.8	896	11 192	18	321
	F	906	N/R					
	P	1035	336					

*Note:* We analysed two trios of an unaffected mother and father and an affected child.

M, Mother; F, Father; P, Proband; T, Trio.

**Table 5.** Results of secure INTERSECTION operation on real patient data

T	Family member	#Rare variants	#Common variants	Protection quotient	Offline phase		Online phase	
					Comm. (MiB)	Time (ms)	Comm. (MiB)	Time (ms)
1	M	860						
	F	967						
	P	1053	29	1-174/5883=97.0	1344	11 572	25	281
2	M	1062						
	F	906						
	P	1035						

*Note*: We analysed six individuals in two trios. Secure INTERSECTION operation found 29 rare variants that are seen in 6 individuals. 6 × 29 rare variants were revealed while keeping the remaining 5709 variants private.

M, Mother; F, Father; P, Proband; T, Trio.

We tested our COMPHET operation on the pigo.vcf file (a family with two daughters affected by Mabry syndrome) provided in the study by Kamphans and Krawitz (2012). We used VCF Explorer (Akgün and Demirci, 2017) for selecting the variants that have less than 5% population allele frequency in any of the subpopulations defined in ESP6500_MAF (NHLBI GO Exome Sequencing Project) and 1000_Genome_MAF (The 1000 Genomes Project Consortium, 2010) and mutation type of missense, synonymous, splicing, frameshift and non-frameshift. Our method outputs 409 variants on 111 genes including the PIGO gene.

MAX, SETDIFF and INTERSECTION operations may be insufficient to find candidate genes and variations that cause the disease because they assume that all participating individuals have the same genotype. Our privacy preserving analyses based on our inheritance models allow researchers to conduct more accurate analysis. As shown in Table 3, MAX operation finds the disease-causing variant and gene in third place, although variants with population allele frequency of less than 1% are selected. Our RECESSIVE operation accurately identifies the disease-causing recessive variant and gene, although variants with a population allele frequency of less than 5% are selected. We ran all experiments on real data in the WAN setting.

### 4.2 Experiments on synthetic data

**Fig. 4.**
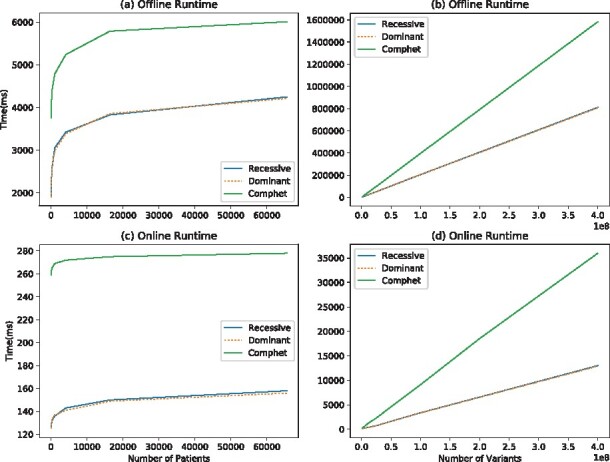
We measure the execution time of our RECESSIVE, DOMINANT and COMPHET operations with respect to an increase in the number of patients and with respect to an increase in the number of variants. (**a**, **c**) The execution time increases logarithmically with the increase in the number of patients. (**b**, **d**) The execution time increases linearly with the increase in the number of variants

**Fig. 5.**
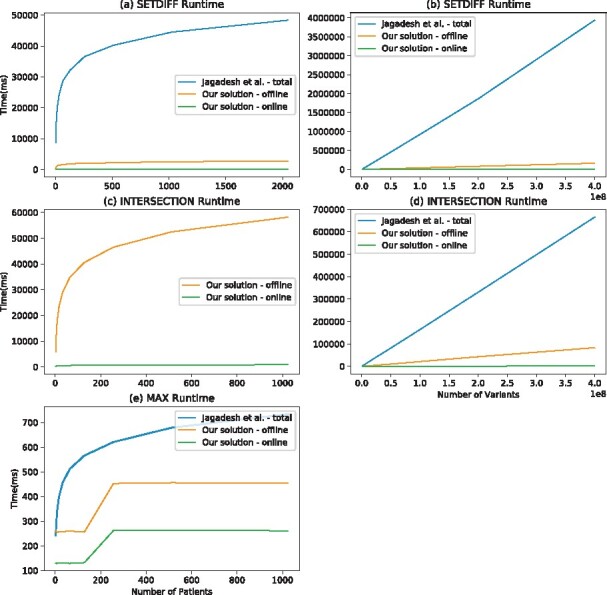
(**a**, **b**) The execution time our SETDIFF operation is much less than Jagadeesh *et al.*’s protocols. It increases linearly as the number of variants increases. In addition, as the number of patients increases, it increases logarithmically. It takes 149 ms to execute the online phase of the SETDIFF operation for 2048 patients and 1 000 000 variants. The execution time of the online phase becomes 3509 ms with three patients and 400 000 000 variants. (**c**, **d**) Our INTERSECTION operation is more efficient than Jagadeesh *et al.*’s protocol in terms of runtime. The execution time increases linearly as the number of variants increases. The online phase of the INTERSECTION operation takes 1951 ms with 2 patients and 400 000 000 variants. The execution time of our INTERSECTION operation increases logarithmically with an increase in the number of patients. Our protocol performs the online phase of the INTERSECTION operation in 847 ms for 1024 patients and 28 000 000 variants. We could not compare our INTERSECTION operation with Jagadeesh *et al.*’s implementation because Jagadeesh *et al.*’s protocol cannot perform the INTERSECTION operation with more than two patients. (**e**) The offline phase of MAX operation performs much better in terms of running time. Furthermore, the online time of our protocol has remarkable advantage over Jagadeesh *et al.*’s (2017) solution. Our protocol performs the MAX operation at most 264 ms for the number of patients up to 65 535. The number of arithmetic-to-Boolean conversions in SETDIFF and INTERSECTION operations are two and one, respectively

In Table 1, we theoretically compare our methods, which are designed using both arithmetic and Boolean gates, and the methods that perform the same processes but designed only with Boolean gates in terms of the number of high-level operations. In our experiments on synthetic data, we measure the performance of our methods with respect to an increasing number of variants and patients in terms of round complexity, circuit size, computation time and communication cost. Thus, we provide a more informative overview of the actual costs of our methods as a function of the input size. In Figure 4, we show the runtimes of the offline and online phase of RECESSIVE, DOMINANT and COMPHET operations for varying patient count and variant count. In Figure 5, we compare our MAX, SETDIFF and INTERSECTION methods with those of Jagadeesh *et al.* (2017). We present how the runtimes of our methods scale with the increase in the number of patients and variants. Details of these experiments are given in the Supplementary Tables S1–S11. In all cases, the circuit size (number of AND gates), the communication costs and the runtimes (offline/online) increase logarithmically with the number of patients and linearly with the number of variants.

We tested the A2B method of the ABY framework that provides conversion from arithmetic sharing to Boolean sharing in the LAN/WAN setting (see Supplementary Table S12). The offline and online runtimes, the number of AND gates and communication cost increase linearly with respect to the increasing number of variants and increase logarithmically with respect to the increasing number of patients. The number of arithmetic to Boolean conversions in RECESSIVE, DOMINANT, COMPHET, SETDIFF and INTERSECTION operations are 2, 2, 3, 2 and 1, respectively. The conversion of an arithmetic share of a vector with a length of 28 000 000 representing a varying number of patients in the range from 2 to 255 into a Boolean share takes 140 251 and 102 755 ms for offline and online phases, respectively. If we use the A2B method of the ABY framework instead of our linear conversion method, the offline/online execution of the RECESSIVE, DOMINANT, COMPHET, SETDIFF and INTERSECTION methods will take 280 502 ms/205 510 ms, 280 502 ms/205 510 ms, 420 753 ms/308 265 ms, 280 502 ms/205 510 ms and 140 251 ms/102 755 ms more time, respectively. The difference in the execution time of the operations in which our conversion method is used and the operations in which the A2B method is used increases logarithmically with the number of patients and linearly with the number of variants. This shows the efficiency of our conversion method in terms of computation and communication cost.

## 5 Conclusion

Today, rare disease analyses are usually carried out without taking any security measures for participating individuals. Thus, individuals’ genomic data are disclosed to researchers who evaluate the analysis. In this work, we have proposed new privacy-preserving methods to analyse multiple genomic data for finding disease-causing mutations. In our solution, secure computation is performed by two non-colluding proxy servers and all security and privacy measures are taken for the privacy of participants. Only variants and genes obtained at the end of the analysis are disclosed to researchers. The performance of the proposed solution is promising for real-life applications. We have used a special way of converting arithmetic shared zero values to Boolean-shared zero values locally. Thus, we have benefited from the efficient and fast operation of non-linear gates in Boolean circuits. The performance of our methods scale logarithmically in the number of individuals, contributing to the analysis. Experimental results demonstrated that the execution times of the RECESSIVE, DOMINANT and COMPHET with 1 000 000 variants and 65 536 patients are 158, 156 and 278 ms, respectively. Furthermore, the execution times of the MAX with 20 000 genes and 1024 patients, SETDIFF with 1 000 000 variants and 2048 patients, and INTERSECTION with 400 000 000 variants and 2 patients are 261, 149 and 1951 ms, respectively. Our methods are the first privacy-preserving protocols for analyzing genomic data using inheritance models to find disease-causing mutations.

## Supplementary Material

btaa641_Supplementary_Data
