# Supplementary material for: Identifying disease-causing mutations with privacy protection
Source: Bioinformatics. 2020 Jul 19;36(21):5205–13. doi: 10.1093/bioinformatics/btaa641 (PMC7850099; doi:10.1093/bioinformatics/btaa641)
Supplement: btaa641_Supplementary_Data [file btaa641_supplementary_data.pdf]

Supplementary for Identifying Disease-Causing Mutations with Privacy Protection

Mete Akgün, Ali Burak Ünal, Bekir Ergüner,
Nico Pfeifer and Oliver Kohlbacher

June 16, 2020

1 Introduction

In this supplementary document, we describe some parts that we do not provide in the main article due to the page limit. We give the algorithmic explanations of our methods. We show the results of our experiments on synthetic data.

2 Operations with Boolean Gates

In this section, we show how RECESSIVE, DOMINANT, COMPHET, MAX, SETDIFF and INTERSECTION operations can be implemented with only boolean gates. With the following figures, we show that the number of non-linear gates required to implement these operations with the boolean circuit increases linearly with the number of participants.

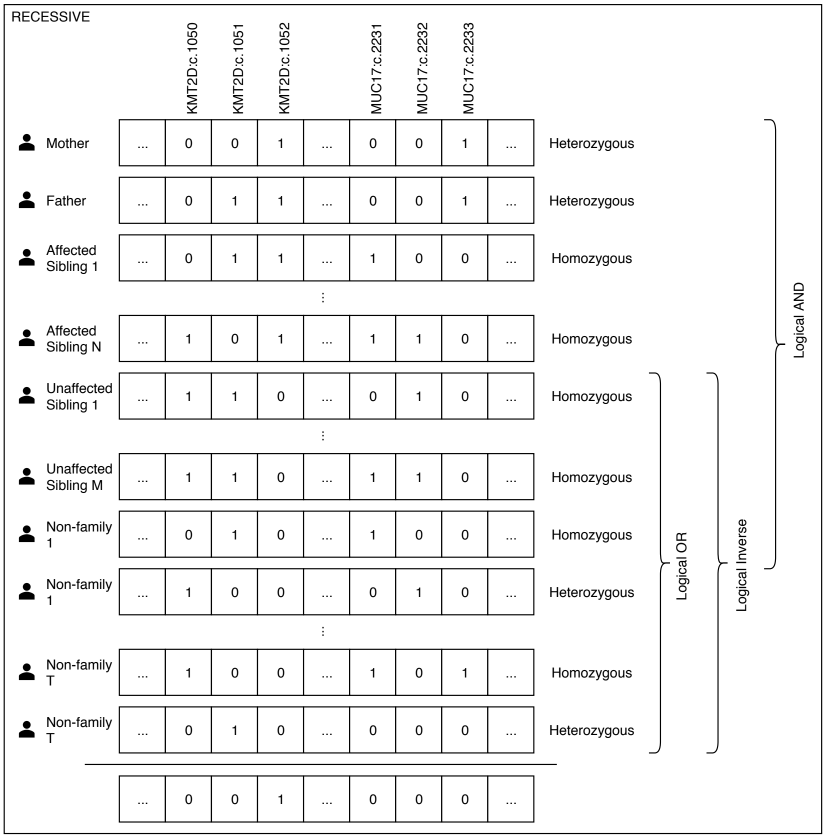

Figure 1: Secure RECESSIVE operation

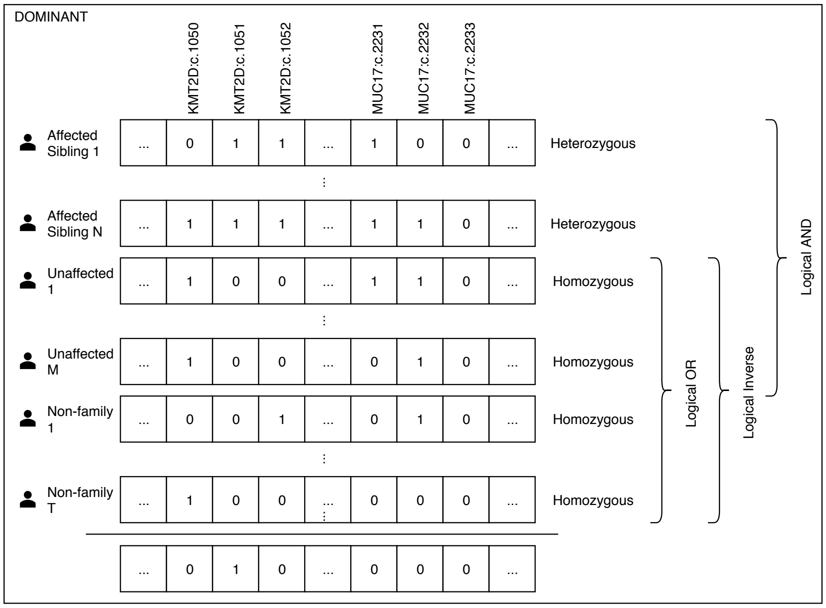

Figure 2: Secure DOMINANT operation

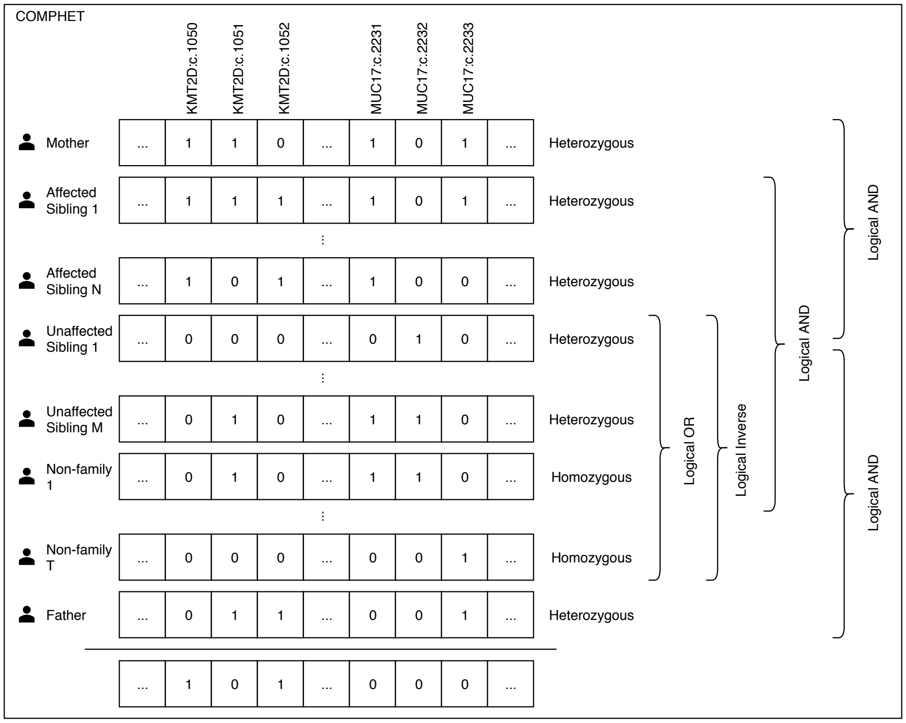

Figure 3: Secure COMPHET operation

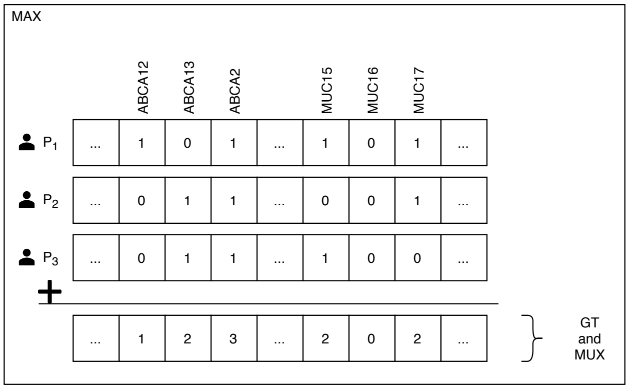

Figure 4: Secure MAX operation

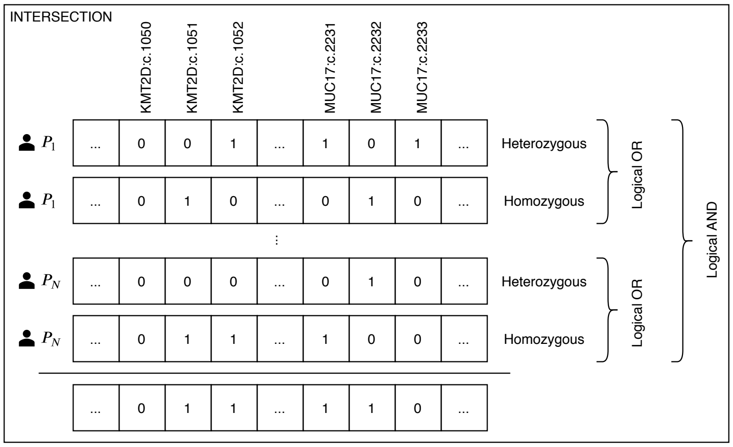

Figure 5: Secure INTERSECTION operation

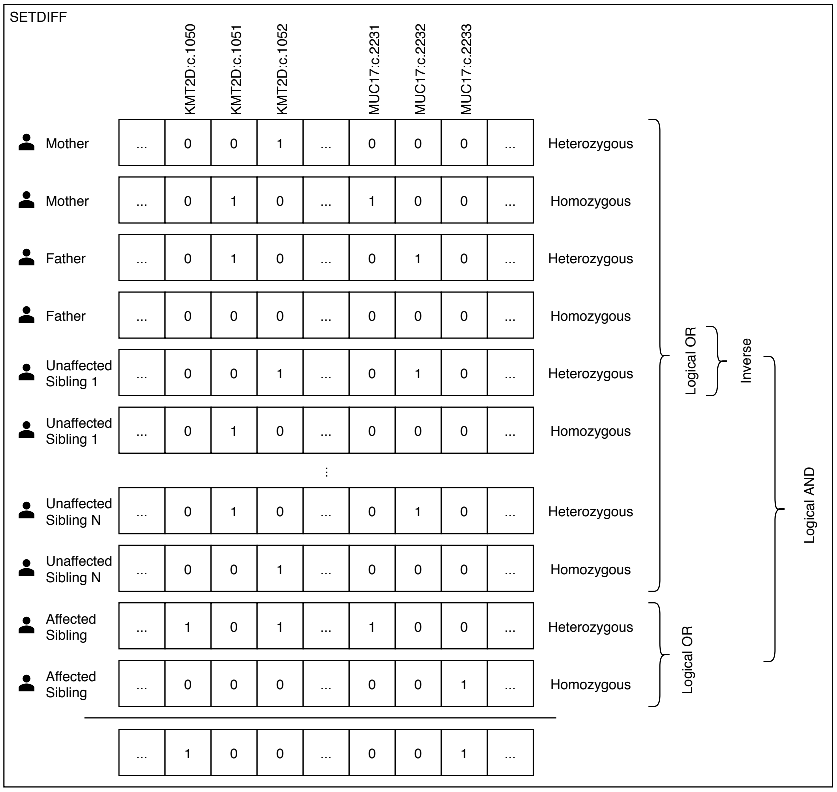

Figure 6: Secure SETDIFF operation

3 Algorithms

In this section, we give the algorithmic descriptions of our privacy-preserving methods implemented with both arithmetic and boolean gates.

3.1 Notations

We denote a shared value x with $\langle x\rangle_a^b$. $b \in \{A, B\}$ indicates the sharing type where A denotes Arithmetic Sharing and B denotes Boolean Sharing. $a \in \{0, 1\}$ is a index of the computing party. We use $\oplus, \wedge, |$ and $=$ for XOR, AND, OR and EQ operations respectively. $\langle x\rangle_a^b \leftarrow \{y\}$ initializes a shared vector whose all elements equals to y and size equals to the size of the variant or the gene vector. $\langle x\rangle_a^b \leftarrow y$ initializes a shared value equals to y. If $\langle x\rangle_a^b$ is a shared vector of random values, $\langle x[i]\rangle_a^b$ is the i-th element of $\langle x\rangle_a^b$. l is the bit length of the shares.

Algorithm $\texttt{A2B}_L\texttt{()}$

input : $\langle v\rangle_t^A$, p, $\langle v\rangle_t^A$ is a vector of random values, n is the size of $\langle v\rangle_t^A$ and $t \in \{0, 1\}$ is the index of the proxy server

output: $\langle v\rangle_t^B$, $\langle v\rangle_t^B$ is a vector of random values

$\langle v\rangle_t^B \leftarrow \langle v\rangle_t^A$

if $t = 0$ **then**

  $tmp \leftarrow \{2^l\}$

  $\langle v\rangle_t^B \leftarrow (tmp - \langle v\rangle_t^A) \bmod 2^l$ `/* local computation */`

Algorithm 1: Arithmetic to Boolean Conversion of Zero Values

3.1.1 RECESSIVE operation on Variant Vectors

In this operation, a researcher wants to test the recessive inheritance model to find disease-causing variants. In the recessive model, the mother and the father are heterozygous carriers and the affected siblings have homozygous variants. If there are unaffected siblings that are considered as heterozygous carriers or homozygous reference. Non-family individuals included in the analysis are considered as homozygous reference. Figure 1 shows how to perform the RECESSIVE operation on variant vectors with boolean operators. The detailed description of our privacy-preserving RECESSIVE operation is given in Algorithm 2.

Algorithm `Recessive()`

input : $\langle v_m\rangle_t^A, \langle v_f\rangle_t^A, (\langle v_a^1\rangle_t^A, ..., \langle v_a^p\rangle_t^A), (\langle v_u^1\rangle_t^A, ..., \langle v_u^r\rangle_t^A), (\langle v_{oh}^1\rangle_t^A, ..., \langle v_{oh}^s\rangle_t^A), (\langle v_{oe}^1\rangle_t^A, ..., \langle v_{oe}^s\rangle_t^A)$, $\langle v_m\rangle_t^A$,$\langle v_f\rangle_t^A$ and $\langle v_{oe}^i\rangle_t^A$ are heterozygous variant vectors, $\langle v_a^i\rangle_t^A$, $\langle v_u^i\rangle_t^A$ and $\langle v_{oh}^i\rangle_t^A$ are homozygous variant vectors, p is the number of affected siblings, r is the number of unaffected siblings, s is the number of others, $t \in \{0,1\}$ is the index of the proxy server

output: $\langle a\rangle_t^B$, a is a vector that gives the locations on which the father and the mother have heterozygous variants, all affected siblings have homozygous variants, all unaffected siblings have non-homozygous variants and, others have no variants, the locations are marked with one

$\langle a\rangle_t^A \leftarrow \{0\}$
for $i \leftarrow 1$ ***to*** p **do** `/* find common homozygous variants of all affected siblings */`
  $\langle a\rangle_t^A \leftarrow (\langle a\rangle_t^A + \langle v_a^i\rangle_t^A) \bmod 2^l$ `/* local computation */`
$\langle a\rangle_t^A \leftarrow (\langle a\rangle_t^A + \langle v_m\rangle_t^A) \bmod 2^l$ `/* local computation */`
$\langle a\rangle_t^A \leftarrow (\langle a\rangle_t^A + \langle v_f\rangle_t^A) \bmod 2^l$ `/* local computation */`
if $t = 0$ **then**
  $tmp \leftarrow \{p+2\}$
  $\langle a\rangle_t^A \leftarrow (\langle a\rangle_t^A - tmp) \bmod 2^l$ `/* local computation, a is a vector that gives the locations on which all affected siblings have homozygous variants and the mother and the father have heterozygous variants, the locations are marked with zero */`
$\langle o\rangle_t^A \leftarrow \{0\}$
for $i \leftarrow 1$ ***to*** r **do** `/* find common non-homozygous variants of unaffected siblings */`
  $\langle o\rangle_t^A \leftarrow (\langle o\rangle_t^A + \langle v_u^i\rangle_t^A) \bmod 2^l$ `/* local computation */`
for $i \leftarrow 1$ ***to*** s **do** `/* find common non-homozygous and non-heterozygous variants of others */`
  $\langle o\rangle_t^A \leftarrow (\langle o\rangle_t^A + \langle v_{oh}^i\rangle_t^A) \bmod 2^l$ `/* local computation */`
  $\langle o\rangle_t^A \leftarrow (\langle o\rangle_t^A + \langle v_{oe}^i\rangle_t^A) \bmod 2^l$ `/* local computation */`
`/* o is a vector that gives the locations on which all unaffected siblings have non-homozygous variants and others have no variants, the locations are marked with zero */`
$\langle a\rangle_t^B \leftarrow A2B_L(\langle a\rangle_t^A)$; $\langle o\rangle_t^B \leftarrow A2B_L(\langle o\rangle_t^A)$ `/* arithmetic → boolean (local computation) */`
$\langle z\rangle_t^B \leftarrow \{0\}$
$\langle a\rangle_t^B \leftarrow \langle a\rangle_t^B = \langle z\rangle_t^B$; $\langle o\rangle_t^B \leftarrow \langle o\rangle_t^B = \langle z\rangle_t^B$ `/* find location of zeros */`
$\langle a\rangle_t^B \leftarrow \langle a\rangle_t^B \wedge \langle o\rangle_t^B$

Algorithm 2: Secure Recessive Operation

3.1.2 DOMINANT operation on Variant Vectors

In this operation, the affected siblings have a single copy of a variant. Thus the parentship information is not important. If there are unaffected siblings they are considered as heterozygous carriers or homozygous reference. Non-family individuals included in the analysis are considered as heterozygous carriers or homozygous reference. Figure 2 shows how to perform the DOMINANT operation on variant vectors with boolean operators. The detailed description of our privacy-preserving DOMINANT operation is given in Algorithm 3.

Algorithm `Dominant()`

input : $(\langle v_a^1\rangle_t^A, ..., \langle v_a^p\rangle_t^A), (\langle v_{uh}^1\rangle_t^A, ..., \langle v_{uh}^r\rangle_t^A), (\langle v_{ue}^1\rangle_t^A, ..., \langle v_{ue}^r\rangle_t^A), (\langle v_{oh}^1\rangle_t^A, ..., \langle v_{oh}^s\rangle_t^A), (\langle v_{oe}^1\rangle_t^A, ..., \langle v_{oe}^s\rangle_t^A)$, $\langle v_{ue}^i\rangle_t^A$ and $\langle v_{oe}^i\rangle_t^A$ are heterozygous variant vectors, $\langle v_a^i\rangle_t^A$, $\langle v_{uh}^i\rangle_t^A$ and $\langle v_{oh}^i\rangle_t^A$ are homozygous variant vectors, p is the number of affected siblings, r is the number of unaffected siblings, s is the number of others, $t \in \{0,1\}$ is the index of the proxy server

output: $\langle a\rangle_t^B$, a is a vector that gives the locations on which all affected siblings have heterozygous variants and, all unaffected siblings and others have homozygous reference variants; the locations are marked with one

$\langle a\rangle_t^A \leftarrow \{0\}$
for $i \leftarrow 1$ ***to*** p **do** `/* find common heterozygous variants of all affected siblings */`
    $\langle a\rangle_t^A \leftarrow (\langle a\rangle_t^A + \langle v_a^i\rangle_t^A) \bmod 2^l$ `/* local computation */`
if $t = 0$ **then**
    $tmp \leftarrow \{p\}$
    $\langle a\rangle_t^A \leftarrow (\langle a\rangle_t^A - tmp) \bmod 2^l$ `/* local computation;` a `is a vector that gives the locations on which all affected siblings have heterozygous variants; the locations are marked with zero */`
$\langle o\rangle_t^A \leftarrow \{0\}$
for $i \leftarrow 1$ ***to*** r **do** `/* find common homozygous reference variants of unaffected siblings */`
    $\langle o\rangle_t^A \leftarrow (\langle o\rangle_t^A + \langle v_{uh}^i\rangle_t^A) \bmod 2^l$ `/* local computation */`
    $\langle o\rangle_t^A \leftarrow (\langle o\rangle_t^A + \langle v_{ue}^i\rangle_t^A) \bmod 2^l$ `/* local computation */`
for $i \leftarrow 1$ ***to*** s **do** `/* find common homozygous reference variants of others */`
    $\langle o\rangle_t^A \leftarrow (\langle o\rangle_t^A + \langle v_{oh}^i\rangle_t^A) \bmod 2^l$ `/* local computation */`
    $\langle o\rangle_t^A \leftarrow (\langle o\rangle_t^A + \langle v_{oe}^i\rangle_t^A) \bmod 2^l$ `/* local computation */`
`/*` o `is a vector that gives the locations on which all unaffected siblings and others have homozygous reference variants; the locations are marked with zero */`
$\langle a\rangle_t^B \leftarrow A2B_L(\langle a\rangle_t^A)$; $\langle o\rangle_t^B \leftarrow A2B_L(\langle o\rangle_t^A)$ `/* arithmetic` $\rightarrow$ `boolean (local computation) */`
$\langle z\rangle_t^B \leftarrow \{0\}$
$\langle a\rangle_t^B \leftarrow \langle a\rangle_t^B = \langle z\rangle_t^B$; $\langle o\rangle_t^B \leftarrow \langle o\rangle_t^B = \langle z\rangle_t^B$ `/* find location of zeros */`
$\langle a\rangle_t^B \leftarrow \langle a\rangle_t^B \wedge \langle o\rangle_t^B$

Algorithm 3: Secure Dominant Operation

3.1.3 COMPHET operation on Variant Vectors

The individual having a compound heterozygous mutation should have at least two heterozygous mutations in a given gene, one from the mother and the other from the father. The unaffected siblings are considered as homozygous carriers or homozygous reference. Non-family individuals included in the analysis are considered as heterozygous carriers or homozygous reference. Figure 3 shows how to perform the COMPHET operation on variant vectors with boolean operators. The detailed description of our privacy-preserving COMPHET operation is given in Algorithm 4.

Algorithm `CompHet()`

input : $\langle v_m \rangle_t^A, \langle v_f \rangle_t^A, (\langle v_a^1 \rangle_t^A, ..., \langle v_a^p \rangle_t^A), (\langle v_u^1 \rangle_t^A, ..., \langle v_u^r \rangle_t^A), (\langle v_o^1 \rangle_t^A, ..., \langle v_o^s \rangle_t^A)$, $\langle v_m \rangle_t^A$, $\langle v_f \rangle_t^A$, $\langle v_a^i \rangle_t^A$, $\langle v_u^i \rangle_t^A$ and $\langle v_o^i \rangle_t^A$ are heterozygous variant vectors, p is the number of affected siblings, r is the number of unaffected siblings, s is the number of others, $t \in \{0, 1\}$ is the index of the proxy server

output: $\langle m \rangle_t^B, \langle f \rangle_t^B$, m and f are gene vectors that gives only the location of compound heterozygous variants; the locations are marked with one

$\langle a \rangle_t^A \leftarrow \{0\}$
for $i \leftarrow 1$ ***to*** p **do** `/* find common heterozygous variants of all affected siblings */`
  $\langle a \rangle_t^A \leftarrow (\langle a \rangle_t^A + \langle v_a^i \rangle_t^A) \bmod 2^l$ `/* local computation */`
$\langle m \rangle_t^A \leftarrow (\langle a \rangle_t^A + \langle v_m \rangle_t^A) \bmod 2^l$ `/* local computation */`
$\langle f \rangle_t^A \leftarrow (\langle a \rangle_t^A + \langle v_f \rangle_t^A) \bmod 2^l$ `/* local computation */`
$tmp \leftarrow \{p+1\}$
if $t = 0$ **then**
  $\langle m \rangle_t^A \leftarrow (\langle m \rangle_t^A - tmp) \bmod 2^l$ `/* local computation;` m `is a vector that gives the locations on which all affected siblings and the mother have non-heterozygous variants; the locations are marked with zero */`
  $\langle f \rangle_t^A \leftarrow (\langle f \rangle_t^A - tmp) \bmod 2^l$ `/* local computation;` f `is a vector that gives the locations on which all affected siblings and the father have non-heterozygous variants; the locations are marked with zero */`
$\langle o \rangle_t^A \leftarrow \{0\}$
for $i \leftarrow 1$ ***to*** r **do** `/* find common non-heterozygous variants of all unaffected siblings */`
  $\langle o \rangle_t^A \leftarrow (\langle o \rangle_t^A + \langle v_u^i \rangle_t^A) \bmod 2^l$ `/* local computation */`
for $i \leftarrow 1$ ***to*** s **do** `/* find common non-heterozygous variants of others */`
  $\langle o \rangle_t^A \leftarrow (\langle o \rangle_t^A + \langle v_o^i \rangle_t^A) \bmod 2^l$ `/* local computation */`
`/*` o `is a vector that gives the locations on which all unaffected siblings and others have non-heterozygous variants; the locations are marked with zero */`
$\langle m \rangle_t^B \leftarrow A2B_L(\langle m \rangle_t^A)$; $\langle f \rangle_t^B \leftarrow A2B_L(\langle f \rangle_t^A)$; $\langle o \rangle_t^B \leftarrow A2B_L(\langle o \rangle_t^A)$ `/* arithmetic` $\rightarrow$ `boolean (local computation) */`
$\langle z \rangle_t^B \leftarrow \{0\}$
$\langle m \rangle_t^B \leftarrow \langle m \rangle_t^B = \langle z \rangle_t^B$; $\langle f \rangle_t^B \leftarrow \langle f \rangle_t^B = \langle z \rangle_t^B$; $\langle o \rangle_t^B \leftarrow \langle o \rangle_t^B = \langle z \rangle_t^B$ `/* find location of zeros */`
$\langle m \rangle_t^B \leftarrow \langle m \rangle_t^B \wedge \langle o \rangle_t^B$ `/*` m `is a vector that gives the locations of heterozygous variants seen in the mother and all affected siblings and, not seen in all unaffected siblings and others; the locations are marked with one */`
$\langle f \rangle_t^B \leftarrow \langle f \rangle_t^B \wedge \langle o \rangle_t^B$ `/*` f `is a vector that gives the locations of heterozygous variants seen in the father and all affected siblings and, not seen in all unaffected siblings and others; the locations are marked with one */`
$\langle tmp \rangle_t^B \leftarrow \langle m \rangle_t^B$
$\langle m \rangle_t^B \leftarrow \langle m \rangle_t^B \wedge \overline{\langle f \rangle_t^B}$ `/* eliminates common heterozygous variants seen in the parents */`
$\langle f \rangle_t^B \leftarrow \langle f \rangle_t^B \wedge \overline{\langle tmp \rangle_t^B}$ `/* eliminates common heterozygous variants seen in the parents */`
$\langle m_c \rangle_t^B \leftarrow \{0\}$; $\langle f_c \rangle_t^B \leftarrow \{0\}$; $k \leftarrow 0$
`/* If the gene has at least one heterozygous variant, fill all the locations in the vector` z `corresponding to variants on the gene with 1, otherwise, fill with 0 */`
foreach *gene* $\langle g_m^i \rangle_t^B \in \langle m \rangle_t^B$ *and gene* $\langle g_f^i \rangle_t^B \in \langle f \rangle_t^B$ **do**
  $\langle tmp_m \rangle_t^B \leftarrow 0$, $\langle tmp_f \rangle_t^B \leftarrow 0$
  foreach *variant* $\langle s_m^j \rangle_t^B \in \langle g_m^i \rangle_t^B$ *and variant* $\langle s_f^j \rangle_t^B \in \langle g_f^i \rangle_t^B$ **do**
    $\langle tmp_m \rangle_t^B \leftarrow \langle tmp_m \rangle_t^B | \langle s_m^j \rangle_t^B$; $\langle tmp_f \rangle_t^B \leftarrow \langle tmp_f \rangle_t^B | \langle s_f^j \rangle_t^B$
  foreach *variant* $\langle s_m^j \rangle_t^B \in \langle g_m^i \rangle_t^B$ *and variant* $\langle s_f^j \rangle_t^B \in \langle g_f^i \rangle_t^B$ **do**
    $\langle m_c[k] \rangle_t^B \leftarrow \langle tmp_m \rangle_t^B$; $\langle f_c[k] \rangle_t^B \leftarrow \langle tmp_f \rangle_t^B$
    $k \leftarrow k + 1$
$\langle m \rangle_t^B \leftarrow \langle m \rangle_t^B \wedge \langle f_c \rangle_t^B$ `/* If the father does not have any heterozygous variants in a gene, set the locations corresponding to the variants on this gene in the mother's vector to 0. */`
$\langle f \rangle_t^B \leftarrow \langle f \rangle_t^B \wedge \langle m_c \rangle_t^B$ `/* If the mother does not have any heterozygous variants in a gene, set the locations corresponding to the variants on this gene in the father's vector to 0. */`

Algorithm 4: Secure CompHet Operation

3.1.4 MAX operation on Gene Vectors

In this operation, a researcher is interested in a cohort of individuals with the same disease or phenotype. He or she wants to find the most commonly mutated genes in the cohort which are potentially associated with the disease. The gene vector indicates whether the genes have rare variants. Therefore, the sum of the gene vectors of the individuals in the cohort allows the researcher to find the most mutated genes. MAX operation with boolean operators is illustrated in Figure 4. The detailed description of our privacy-preserving MAX operation is given in Supplementary Algorithm 5.

Algorithm `SecureMax()`

  input : $(\langle v_0 \rangle_t^A, ..., \langle v_n \rangle_t^A)$, $\langle v_i \rangle_t^A$ is a gene vector, n is the number of patients, $t \in \{0, 1\}$ is the index of the proxy server

  output: $\langle m \rangle_t^B$, m is a gene vector that gives the locations of most mutated genes; the locations are marked with one

  $\langle r \rangle_t^A \leftarrow \{0\}$

  for $i \leftarrow 1$ ***to*** n **do**

    $\langle r \rangle_t^A \leftarrow (\langle r \rangle_t^A + \langle v_i \rangle_t^A) \bmod 2^l$ `/* local computation */`

  $\langle r \rangle_t^B \leftarrow \text{A2B}(\langle r \rangle_t^A)$ `/* arithmetic → boolean (communication and cryptographic operations) */`

  $\langle max \rangle_t^B \leftarrow 0$

  foreach *gene* $\langle g \rangle_t^B \in \langle r \rangle_t^B$ **do**

    $\langle tmp \rangle_t^B \leftarrow \langle g \rangle_t^B > \langle max \rangle_t^B$

    $\langle max \rangle_t^B \leftarrow \text{MUX}(\langle g \rangle_t^B, \langle max \rangle_t^B, \langle tmp \rangle_t^B)$

  $\langle m \rangle_t^B \leftarrow \{\langle max \rangle_t^B\}$

  $\langle m \rangle_t^B \leftarrow \langle m \rangle_t^B \wedge \langle r \rangle_t^B$

Algorithm 5: Secure Max Operation

3.1.5 SETDIFF operation on Variant Vectors

In this operation, a researcher wants to find the variants that cause a disease in a family. He or she wants to analyze an affected individual and unaffected individuals in the family. Non-family members can be included to the analysis for more accurate results. For SETDIFF operation, we need to obtain a bit vector that shows the ownership of information of variants in the given variant list. We can obtain this bit vector by adding the vector of heterozygous variants to the vector of homozygous variants. SETDIFF operation with boolean operators is illustrated in Figure 6. The detailed description of our privacy-preserving SETDIFF operation is given in Algorithm 6.

Algorithm `SecureSetDiff()`

input : $\langle v_{mh}\rangle_t^A, \langle v_{me}\rangle_t^A, \langle v_{fh}\rangle_t^A, \langle v_{fe}\rangle_t^A, (\langle v_{ah}^1\rangle_t^A, ..., \langle v_{ah}^p\rangle_t^A), (\langle v_{ae}^1\rangle_t^A, ..., \langle v_{ae}^p\rangle_t^A), (\langle v_{uh}^1\rangle_t^A, ..., \langle v_{uh}^r\rangle_t^A), (\langle v_{ue}^1\rangle_t^A, ..., \langle v_{ue}^r\rangle_t^A)$, $\langle v_{mh}\rangle_t^A$,$\langle v_{fh}\rangle_t^A$, $\langle v_{ah}^i\rangle_t^A$ and $\langle v_{uh}^i\rangle_t^A$ are homozygous variant vectors, $\langle v_{me}\rangle_t^A$, $\langle v_{fe}\rangle_t^A$, $\langle v_{ae}^i\rangle_t^A$ and $\langle v_{ue}^i\rangle_t^A$ are heterozygous variant vectors, p is the number of affected siblings, r is the number of unaffected siblings, $t \in \{0, 1\}$ is the index of the proxy server

output: $\langle a\rangle_t^B$, a is a variant vector that gives the locations on which all affected siblings have variants and the mother, the father and all unaffected siblings have no variant; the locations are marked with one

$\langle a\rangle_t^A \leftarrow \{0\}$
for $i \leftarrow 1$ ***to*** p **do** `/* find common variants of all affected siblings */`
  $\langle a\rangle_t^A \leftarrow (\langle a\rangle_t^A + \langle v_{ah}^i\rangle_t^A) \bmod 2^l$ `/* local computation */`
  $\langle a\rangle_t^A \leftarrow (\langle a\rangle_t^A + \langle v_{ae}^i\rangle_t^A) \bmod 2^l$ `/* local computation */`
if $t = 0$ **then**
  $\langle tmp\rangle_t^A \leftarrow \{p\}$
  $\langle a\rangle_t^A \leftarrow (\langle a\rangle_t^A - \langle tmp\rangle_t^A) \bmod 2^l$ `/* local computation;` a `is a vector that gives the locations on which all affected siblings have variants; the locations are marked with zero */`
$\langle o\rangle_t^A \leftarrow \{0\}$
for $i \leftarrow 1$ ***to*** r **do** `/* find common locations where unaffected siblings have no variant */`
  $\langle o\rangle_t^A \leftarrow (\langle o\rangle_t^A + \langle v_{uh}^i\rangle_t^A) \bmod 2^l$ `/* local computation */`
  $\langle o\rangle_t^A \leftarrow (\langle o\rangle_t^A + \langle v_{ue}^i\rangle_t^A) \bmod 2^l$ `/* local computation */`
$\langle o\rangle_t^A \leftarrow (\langle o\rangle_t^A + \langle v_{mh}\rangle_t^A) \bmod 2^l$ `/* local computation */`
$\langle o\rangle_t^A \leftarrow (\langle o\rangle_t^A + \langle v_{me}\rangle_t^A) \bmod 2^l$ `/* local computation */`
$\langle o\rangle_t^A \leftarrow (\langle o\rangle_t^A + \langle v_{fh}\rangle_t^A) \bmod 2^l$ `/* local computation */`
$\langle o\rangle_t^A \leftarrow (\langle o\rangle_t^A + \langle v_{fe}\rangle_t^A) \bmod 2^l$ `/* local computation */`
`/*` o `is a vector that gives the locations on which all unaffected siblings, the mother and the father have no variant; the locations are marked with zero */`
$\langle a\rangle_t^B \leftarrow A2B_L(\langle a\rangle_t^A)$; $\langle o\rangle_t^B \leftarrow A2B_L(\langle o\rangle_t^A)$ `/* arithmetic` $\rightarrow$ `boolean (local computation) */`
$\langle z\rangle_t^B \leftarrow \{0\}$
$\langle a\rangle_t^B \leftarrow \langle a\rangle_t^B = \langle z\rangle_t^B$; $\langle o\rangle_t^B \leftarrow \langle o\rangle_t^B = \langle z\rangle_t^B$ `/* find location of zeros */`
$\langle a\rangle_t^B \leftarrow \langle a\rangle_t^B \wedge \langle o\rangle_t^B$

Algorithm 6: Secure SetDiff Operation

3.1.6 INTERSECTION operation on Variant Vectors

In this operation, a researcher is interested in a group of the unrelated individuals. He or she wants to find variants seen in all the individuals in this group. We have two variant vectors for each patient. For INTERSECTION operation, we need to obtain a bit vector that shows the ownership of information of variants in the given variant list. We can obtain this bit vector by adding the vector of heterozygous variants to the vector of homozygous variants. Variants that are seen in all the individuals are found by computing logical AND of the variant vectors of all the individuals. INTERSECTION operation is illustrated in Figure 5. The detailed description of our privacy-preserving INTERSECTION operation is given in Algorithm 7.

Algorithm `SecureIntersection()`

input : $(\langle v_h^1 \rangle_t^A, ..., \langle v_h^p \rangle_t^A), (\langle v_e^1 \rangle_t^A, ..., \langle v_e^p \rangle_t^A)$, $\langle v_h^i \rangle_t^A$ is homozygous a variant vector, $\langle v_e^i \rangle_t^A$ is a heterozygous variant vector, p is the number of individuals, $t \in \{0, 1\}$ is the index of the proxy server

output: $\langle a \rangle_t^B$, a is a variant vector that gives the locations on which all individuals have variants; the locations are marked with one

$\langle a \rangle_t^A \leftarrow \{0\}$

for $i \leftarrow 1$ ***to*** p **do** `/* find common variants of all individuals */`

  $\langle a \rangle_t^A \leftarrow (\langle a \rangle_t^A + \langle v_h^i \rangle_t^A) \bmod 2^l$ `/* local computation */`

  $\langle a \rangle_t^A \leftarrow (\langle a \rangle_t^A + \langle v_e^i \rangle_t^A) \bmod 2^l$ `/* local computation */`

if $t = 0$ **then**

  $\langle tmp \rangle_t^A \leftarrow \{p\}$

  $\langle a \rangle_t^A \leftarrow (\langle a \rangle_t^A - \langle tmp \rangle_t^A) \bmod 2^l$ `/* local computation;` a `is a vector that gives the locations on which all individuals have variants; the locations are marked with zero */`

$\langle a \rangle_t^B \leftarrow A2B_L(\langle a \rangle_t^A)$; `/* arithmetic` $\rightarrow$ `boolean (local computation) */`

$\langle z \rangle_t^B \leftarrow \{0\}$

$\langle a \rangle_t^B \leftarrow \langle a \rangle_t^B = \langle z \rangle_t^B$; `/* find location of zeros */`

Algorithm 7: Secure Intersection Operation

4 Experiments on Synthetic Data

In this section, we give the detailed results of our experiments on synthetic data.

Table 1: Results of RECESSIVE operation for varying patient count

				Offline Phase		Online Phase	
#Patients	#Variants	#ANDs	Depth	Comm (MiB)	Time (ms) (LAN/WAN)	Comm (MiB)	Time (ms) (LAN/WAN)
32	1,000,000	$9.0 \cdot 10^6$	5	144	1,434/1,945	2	38/126
64	1,000,000	$1.0 \cdot 10^7$	5	160	1,594/2,201	3	43/128
256	1,000,000	$1.2 \cdot 10^7$	5	192	1,880/2,575	3	51/132
1024	1,000,000	$1.4 \cdot 10^7$	6	224	2,204/3,053	4	57/136
4096	1,000,000	$1.6 \cdot 10^7$	6	256	2,487/3,422	4	64/143
16384	1,000,000	$1.8 \cdot 10^7$	6	288	2,823/3,828	5	68/150
65536	1,000,000	$2.0 \cdot 10^7$	6	320	3,042/4,248	5	74/158

[a] All tests were performed on a mother, a father, 14 unaffected siblings, and 16 unaffected siblings. The number of non-family individuals varies.

Table 2: Results of RECESSIVE operation for varying variant count

				Offline Phase		Online Phase	
#Patients	#Variants	#ANDs	Depth	Comm (MiB)	Time (ms) (LAN/WAN)	Comm (MiB)	Time (ms) (LAN/WAN)
64	1,000,000	$1.0 \cdot 10^7$	5	160	1,594/2,201	3	43/128
64	5,000,000	$5.0 \cdot 10^7$	5	800	7,584/10,415	13	176/227
64	10,000,000	$1.0 \cdot 10^8$	5	1,600	15,712/20,561	26	321/355
64	25,000,000	$2.5 \cdot 10^8$	5	4,000	37,836/51,483	66	633/732
64	50,000,000	$5.0 \cdot 10^8$	5	8,000	74,921/102,720	131	1,258/1,626
64	100,000,000	$1.0 \cdot 10^9$	5	16,000	149,250/205,532	262	2,491/3,373
64	200,000,000	$2.0 \cdot 10^9$	5	32,000	299,088/407,275	525	4,815/6,571
64	400,000,000	$4.0 \cdot 10^9$	5	64,000	597,042/811,533	1,050	9,697/12,997

[a] All tests were performed on a mother, a father, 14 unaffected siblings, 16 unaffected siblings, and 32 non-family individuals.

Table 3: Results of DOMINANT operation for varying patient count

#Patients	#Variants	#ANDs	Depth	Offline Phase		Online Phase	
				Comm (MiB)	Time (ms) (LAN/WAN)	Comm (MiB)	Time (ms) (LAN/WAN)
32	1,000,000	$9.0 \cdot 10^6$	5	144	1,429/1,901	2	39/125
64	1,000,000	$1.0 \cdot 10^7$	5	160	1,607/2,176	3	42/127
256	1,000,000	$1.2 \cdot 10^7$	5	192	1,901/2,623	3	51/133
1024	1,000,000	$1.4 \cdot 10^7$	6	224	2,199/2,988	4	56/137
4096	1,000,000	$1.6 \cdot 10^7$	6	256	2,412/3,387	4	64/141
16384	1,000,000	$1.8 \cdot 10^7$	6	288	2,897/3,856	5	69/149
65536	1,000,000	$2.0 \cdot 10^7$	6	320	3,137/4,212	5	73/156

[a] All tests were performed on 16 unaffected siblings, and 16 unaffected siblings. The number of non-family individuals varies.

Table 4: Results of DOMINANT operation for varying variant count

#Patients	#Variants	#ANDs	Depth	Offline Phase		Online Phase	
				Comm (MiB)	Time (ms) (LAN/WAN)	Comm (MiB)	Time (ms) (LAN/WAN)
64	1,000,000	$1.0 \cdot 10^7$	5	160	1,607/2,237	3	42/130
64	5,000,000	$5.0 \cdot 10^7$	5	800	7,651/10,245	13	181/219
64	10,000,000	$1.0 \cdot 10^8$	5	1,600	15,682/20,788	26	359/340
64	25,000,000	$2.5 \cdot 10^8$	5	4,000	38,133/50,877	66	852/733
64	50,000,000	$5.0 \cdot 10^8$	5	8,000	74,528/103,437	131	1,242/1,611
64	100,000,000	$1.0 \cdot 10^9$	5	16,000	148,871/207,392	262	2,388/3,351
64	200,000,000	$2.0 \cdot 10^9$	5	32,000	300,136/404,288	525	4,737/6,529
64	400,000,000	$4.0 \cdot 10^9$	5	64,000	599,255/807,753	1,050	9,864/12,882

[a] All tests were performed on 16 unaffected siblings, 16 unaffected siblings, and 32 non-family individuals.

Table 5: Results of COMPHET operation for varying patient count

#Patients	#Variants	#ANDs	Depth	Offline Phase		Online Phase	
				Comm (MiB)	Time (ms) (LAN/WAN)	Comm (MiB)	Time (ms) (LAN/WAN)
32	1,000,000	$1.8 \cdot 10^7$	17	289	2,724/3,751	5	111/259
64	1,000,000	$1.9 \cdot 10^7$	17	305	2,885/4,009	5	117/262
256	1,000,000	$2.1 \cdot 10^7$	17	337	3,174/4.390	6	121/265
1024	1,000,000	$2.3 \cdot 10^7$	18	369	3,424/4,785	6	125/269
4096	1,000,000	$2.5 \cdot 10^7$	18	401	3,734/5,241	7	129/272
16384	1,000,000	$2.7 \cdot 10^7$	18	433	4,144/5,788	7	134/275
65536	1,000,000	$2.9 \cdot 10^7$	18	465	4,351/6,008	8	139/278

[a] All tests were performed on a mother, a father, 14 unaffected siblings, and 16 unaffected siblings. The number of non-family individuals varies.

Table 6: Results of COMPHET operation for varying variant count

#Patients	#Variants	#ANDs	Depth	Offline Phase		Online Phase	
				Comm (MiB)	Time (ms) (LAN/WAN)	Comm (MiB)	Time (ms) (LAN/WAN)
64	1,000,000	$1.9 \cdot 10^7$	17	305	2,885/4,009	5	117/262
64	5,000,000	$1.0 \cdot 10^8$	20	1,622	15,003/20,704	27	470/558
64	10,000,000	$2.0 \cdot 10^8$	21	3,244	29,612/40,639	53	911/1,124
64	25,000,000	$4.9 \cdot 10^8$	22	7,849	71,968/98,693	129	2,070/2,293
64	50,000,000	$9.8 \cdot 10^8$	23	15,697	143,980/198,339	258	4,114/4,607
64	100,000,000	$1.9 \cdot 10^9$	24	31,394	285,177/397,521	517	8,005/9,122
64	200,000,000	$3.8 \cdot 10^9$	25	62,789	569,855/792,378	1,034	15,756/18,544
64	400,000,000	$7.6 \cdot 10^9$	26	125,578	1,127,642/1,583,497	2,069	30,589/35,971

[a] All tests were performed on a mother, a father, 14 unaffected siblings, 16 unaffected siblings, and 32 non-family individuals.

Table 7: Results of MAX operation for varying patient count

		Jagadeesh et al.'s Solution [1]		Our Solution					
						Offline Phase		Online Phase	
#Patients	#Variants	Comm (MiB)	Time (ms)	#ANDs	Depth	Comm (MiB)	Time (ms) (LAN/WAN)	Comm (MiB)	Time (ms) (LAN/WAN)
2	20,000	20	158/243	$4.6 \cdot 10^5$	5	10	234/257	12	115/130
4	20,000	30	228/284	$4.6 \cdot 10^5$	5	10	237/261	12	113/129
8	20,000	41	257/340	$4.6 \cdot 10^5$	5	10	232/255	12	118/129
16	20,000	52	287/397	$4.6 \cdot 10^5$	5	10	235/257	12	117/131
32	20,000	62	312/455	$4.6 \cdot 10^5$	5	10	235/258	12	115/130
64	20,000	73	352/512	$4.6 \cdot 10^5$	5	10	235/260	12	117/129
128	20,000	83	382/566	$4.6 \cdot 10^5$	5	10	233/256	12	114/132
256	20,000	94	422/621	$9.4 \cdot 10^5$	5	22	388/453	23	201/262
512	20,000	104	455/679	$9.4 \cdot 10^5$	5	22	388/456	23	201/264
1,024	20,000	114	493/734	$9.4 \cdot 10^5$	5	22	386/455	23	202/261

Table 8: Results of SETDIFF operation for varying patient count

		Jagadeesh et al.'s Solution [1]		Our Solution					
						Offline Phase		Online Phase	
#Patients	#Variants	Comm (MiB)	Time (ms)	#ANDs	Depth	Comm (MiB)	Time (ms) (LAN/WAN)	Comm (MiB)	Time (ms) (LAN/WAN)
2	1,000,000	656	3,319/8,780	$2.0 \cdot 10^6$	3	33	391/570	0	8/35
4	1,000,000	960	4,574/12.101	$3.0 \cdot 10^6$	4	49	528/776	0	11/47
8	1,000,000	1,264	5,922/16.464	$4.0 \cdot 10^6$	4	65	675/954	1	16/59
16	1,000,000	1,568	7,487/20.182	$5.0 \cdot 10^6$	5	81	826/1,223	1	20/81
32	1,000,000	1,872	9,187/24.260	$6.0 \cdot 10^6$	5	98	972/1,403	1	25/98
64	1,000,000	2,176	10,478/28.644	$7.0 \cdot 10^6$	5	114	1,133/1,571	2	28/113
128	1,000,000	2,480	11,917/32.124	$8.0 \cdot 10^6$	5	130	1,256/1,869	2	33/126
256	1,000,000	2,784	13,286/36.566	$9.0 \cdot 10^6$	6	146	1,406/2,019	2	37/135
512	1,000,000	3,088	14,527/40.248	$1.0 \cdot 10^7$	6	163	1,549/2,190	2	41/139
1,024	1,000,000	3,392	15,874/44.491	$1.1 \cdot 10^7$	6	179	1,693/2,550	3	45/144
2,048	1,000,000	3,696	17,244/48.349	$1.2 \cdot 10^7$	6	195	1,879/2,641	3	48/149

Table 9: Results of SETDIFF operation for varying variant count

		Jagadeesh et al.'s Solution [1]		Our Solution					
						Offline Phase		Online Phase	
#Patients	#Variants	Comm (MiB)	Time (ms)	#ANDs	Depth	Comm (MiB)	Time (ms) (LAN/WAN)	Comm (MiB)	Time (ms) (LAN/WAN)
2	1,000,000	656	3,319/8,780	$2.0 \cdot 10^6$	3	33	391/570	0	8/35
2	5,000,000	3,280	17,598/45,212	$1.0 \cdot 10^7$	3	160	1,578/2,307	3	41/117
2	10,000,000	6,560	33,192/91,743	$2.0 \cdot 10^7$	3	320	3,027/4,294	6	79/165
2	25,000,000	16,400	88,270/229,428	$5.0 \cdot 10^7$	3	800	7,587/10,827	16	195/277
2	50,000,000	32,800	166,306/460,779	$1.0 \cdot 10^8$	3	1,600	14,823/20,847	31	387/473
2	100,000,000	65,600	326,807/923,842	$2.0 \cdot 10^8$	3	3,200	29,481/40,825	63	725/882
2	200,000,000	131,200	642,987/1,863,735	$4.0 \cdot 10^8$	3	6,400	59,572/82,777	125	1,484/1,757
2	400,000,000	262,400	1,364,717/3,938,462	$8.0 \cdot 10^8$	3	12,800	119,348/163,623	250	2,917/3,509

Table 10: Results of INTERSECTION operation for varying variant count

		Jagadeesh et al.'s Solution [1]		Our Solution					
						Offline Phase		Online Phase	
#Patients	#Variants	Comm (MiB)	Time (ms)	#ANDs	Depth	Comm (MiB)	Time (ms) (LAN/WAN)	Comm (MiB)	Time (ms) (LAN/WAN)
2	1,000,000	112	709/1,998	$1.0 \cdot 10^6$	2	16	244/337	0	6/15
2	5,000,000	560	3,232/8,153	$5.0 \cdot 10^6$	2	80	810/1,181	2	31/74
2	10,000,000	1,120	6,606/16,950	$1.0 \cdot 10^7$	2	160	1,533/2,194	4	56/134
2	25,000,000	2,800	21,935/41,635	$2.5 \cdot 10^7$	2	400	3,710/5,268	9	113/191
2	50,000,000	5,600	42,866/81,921	$5.0 \cdot 10^7$	2	800	7,397/10,406	19	206/298
2	100,000,000	11,200	84,533/164,344	$1.0 \cdot 10^8$	2	1,600	14,785/21,388	38	401/536
2	200,000,000	22,400	168,617/330,849	$2.0 \cdot 10^8$	2	3,200	28,911/42,996	75	788/977
2	400,000,000	44,800	337,174/665,402	$4.0 \cdot 10^8$	2	6,400	58,007/83,436	150	1,547/1,951

Table 11: Results of INTERSECTION operation for varying patient count

				Our Solution			
				Offline Phase		Online Phase	
#Patients	#Variants	#ANDs	Depth	Comm (MiB)	Time (ms) (LAN/WAN)	Comm (MiB)	Time (ms) (LAN/WAN)
2	28,000,000	$2.8 \cdot 10^7$	2	448	4,335/5,841	11	148/201
4	28,000,000	$5.6 \cdot 10^7$	3	896	8,518/11,623	18	233/277
8	28,000,000	$8.4 \cdot 10^7$	3	1,344	12,842/17,446	25	325/351
16	28,000,000	$1.1 \cdot 10^8$	4	1,792	17,115/23,110	32	415/429
32	28,000,000	$1.4 \cdot 10^8$	4	2,240	21,886/28,887	39	478/507
64	28,000,000	$1.6 \cdot 10^8$	4	2,688	25,466/34,810	46	535/555
128	28,000,000	$1.9 \cdot 10^8$	4	3,136	29,563/40,614	53	637/666
256	28,000,000	$2.2 \cdot 10^8$	5	3,584	33,770/46,522	60	685/703
512	28,000,000	$2.5 \cdot 10^8$	5	4,032	38,075/52,491	67	718/766
1,024	28,000,000	$2.8 \cdot 10^8$	5	4,480	42,523/58,191	74	785/847

Table 12: Performance of A2B method of ABY Framework for varying patient and variant count

				Offline Phase		Online Phase	
#Patients	#Variants	#ANDs	Depth	Comm (MiB)	Time (ms) (LAN/WAN)	Comm (MiB)	Time (ms) (LAN/WAN)
	1,000,000	$7.0 \cdot 10^6$	7	352	3,449/5,170	388	2,443/3,726
	5,000,000	$3.5 \cdot 10^7$	7	1,760	16,863/25,321	1,940	12,915/18,311
	10,000,000	$7.0 \cdot 10^7$	7	3,520	33,492/49,874	3,880	26,103/36,173
2-255	25,000,000	$1.7 \cdot 10^8$	7	8,800	84,225/124,632	9,700	66,472/91,813
	50,000,000	$3.5 \cdot 10^8$	7	17,600	170,711/250,194	19,400	133,521/182,258
	100,000,000	$7.0 \cdot 10^8$	7	35,200	339,657/498,768	38,800	268,435/361,731
	200,000,000	$1.4 \cdot 10^9$	7	70,400	683,472/999,481	77,600	539,883/719,368
	400,000,000	$2.8 \cdot 10^9$	7	140,800	1,367,688/1,998,865	155,200	1,082,217/1,435,527
	1,000,000	$1.5 \cdot 10^7$	7	736	7,157/10,350	776	3,931/7,784
	5,000,000	$7.5 \cdot 10^7$	7	3,680	34,433/51,721	3,880	18,482/37,920
	10,000,000	$1.5 \cdot 10^8$	7	7,360	69,948/102,546	7,760	37,758/75,823
256-65535	25,000,000	$3.7 \cdot 10^8$	7	18,400	175,925/256,392	19,400	91,275/186,692
	50,000,000	$7.5 \cdot 10^8$	7	36,800	349,964/511,344	38,800	194,233/369,293
	100,000,000	$1.5 \cdot 10^9$	7	73,600	711,372/1,009,341	77,600	390,256/741,892
	200,000,000	$3.0 \cdot 10^9$	7	147,200	1,419,749/2,043,296	155,200	783,106/1,477,739
	400,000,000	$6.0 \cdot 10^9$	7	294,400	2,792,158/4,092,773	310,400	1,569,388/2,911,361

References

[1] Karthik A. Jagadeesh, David J. Wu, Johannes A. Birgmeier, Dan Boneh, and Gill Bejerano. Deriving genomic diagnoses without revealing patient genomes. *Science*, 357(6352):692–695, 2017.
